# Spatial RNA Sequencing Identifies Robust Markers of Vulnerable and Resistant Human Midbrain Dopamine Neurons and Their Expression in Parkinson’s Disease

**DOI:** 10.3389/fnmol.2021.699562

**Published:** 2021-07-08

**Authors:** Julio Aguila, Shangli Cheng, Nigel Kee, Ming Cao, Menghan Wang, Qiaolin Deng, Eva Hedlund

**Affiliations:** ^1^Department of Neuroscience, Karolinska Institutet, Stockholm, Sweden; ^2^Department of Physiology and Pharmacology, Karolinska Institutet, Stockholm, Sweden; ^3^Center for Molecular Medicine, Karolinska University Hospital, Stockholm, Sweden; ^4^Department of Cell and Molecular Biology, Karolinska Institutet, Stockholm, Sweden; ^5^Department of Biochemistry and Biophysics, Stockholm University, Stockholm, Sweden

**Keywords:** human midbrain dopamine neurons, spatial transcriptomics, laser microdissection, RNA sequencing, substantia nigra compacta, ventral tegmental area, Parkinson’s disease

## Abstract

Defining transcriptional profiles of substantia nigra pars compacta (SNc) and ventral tegmental area (VTA) dopamine neurons is critical to understanding their differential vulnerability in Parkinson’s Disease (PD). Here, we determine transcriptomes of human SNc and VTA dopamine neurons using LCM-seq on a large sample cohort. We apply a bootstrapping strategy as sample input to DESeq2 and identify 33 stably differentially expressed genes (DEGs) between these two subpopulations. We also compute a minimal sample size for identification of stable DEGs, which highlights why previous reported profiles from small sample sizes display extensive variability. Network analysis reveal gene interactions unique to each subpopulation and highlight differences in regulation of mitochondrial stability, apoptosis, neuronal survival, cytoskeleton regulation, extracellular matrix modulation as well as synapse integrity, which could explain the relative resilience of VTA dopamine neurons. Analysis of PD tissues showed that while identified stable DEGs can distinguish the subpopulations also in disease, the SNc markers SLIT1 and ATP2A3 were down-regulated and thus appears to be biomarkers of disease. In summary, our study identifies human SNc and VTA marker profiles, which will be instrumental for studies aiming to modulate dopamine neuron resilience and to validate cell identity of stem cell-derived dopamine neurons.

## Introduction

Midbrain dopamine neurons are divided into two major populations, the substantia nigra pars compacta (SNc) and the ventral tegmental area (VTA) (Hedlund and Perlmann, 2009). SNc dopamine neurons project to the dorsolateral striatum (Dahlstroem and Fuxe, 1964) and are severely affected in Parkinson’s Disease (PD) (Damier et al., 1999a,b), while VTA dopamine neurons project to cortical and mesolimbic areas and are more resilient to degeneration (Hedlund and Perlmann, 2009). These neuron populations have been extensively investigated in numerous rodent models (Grimm et al., 2004; Chung et al., 2005; Greene et al., 2005; Bifsha et al., 2014; Poulin et al., 2014), toward the goal of identifying molecular mechanisms that can prevent degeneration or to model disease. Targeted analysis of midbrain dopamine neuron populations has revealed several markers that appear to differentially label SNc e.g., *Aldh1a7*, *Sox6*, *Cbln1*, *Vav3*, *Atp2a3*, and VTA e.g., *Calb1*, *Otx2*, *Crym*, *Cadm1*, and *Marcks* (Damier et al., 1999a; Grimm et al., 2004; Chung et al., 2005; Greene et al., 2005; Di Salvio et al., 2010; Bifsha et al., 2014; Panman et al., 2014; Nichterwitz et al., 2016). Transcriptional analysis of human tissue has largely been limited to SNc (Cantuti-Castelvetri et al., 2007; Reyes et al., 2012) except for our recent small sample cohort to compare SNc and VTA (Nichterwitz et al., 2016). These aforementioned investigations display extensive cross-study variability, resulting in very few reproducible markers either within mouse, rat and human or across different species. Small sample sizes could be a confounding factor of these studies, along with differences in rodent strain backgrounds, methodological differences, or inter-individual variability among human patients.

To reveal cell intrinsic properties underlying the differential vulnerability of SNc and VTA dopamine neurons in PD, a thorough large-scale transcriptional profiling in adult human tissues is required. Such an analysis could also investigate the necessary minimum cohort size, above which lineage specific markers remain stably differentially expressed irrespective of patient selection, an essential requirement for valid study design in variable human populations. Finally, identified differences could also serve as a foundation for the selective *in vitro* derivation of SNc dopamine neurons, which represent the ideal cell type for transplantation in PD (Schultzberg et al., 1984; Haque et al., 1997; Taylor et al., 1999; Thompson et al., 2005; Hedlund and Perlmann, 2009; Kriks et al., 2011; Ganat et al., 2012).

Here we used the spatial transcriptomics method LCM-seq, which combines laser capture microdissection with Smart-seq2 (Picelli et al., 2013) RNA sequencing (Nichterwitz et al., 2016, 2018), to precisely analyze individually isolated SNc and VTA dopamine neurons from 18 human *post-mortem* brains. Using bootstrapping without replacement coupled with DESeq2, we identify 33 markers that were stably differentially expressed between SNc and VTA dopamine neurons. We show that the minimal sample size required to reliably identify these subtype-specific markers in this cohort is eight subjects, which may explain why smaller cohorts have given inconsistent results. Several of the markers identified here have been implicated in PD or other degenerative diseases and thus provide compelling future targets to modulate neuronal vulnerability or to model disease. We also analyzed the regulation of these stable genes in PD patient tissues and found that these markers still define the two subpopulations in end-stage disease and that only two SNc markers, SLIT1 and ATP2A3, were severely down-regulated in PD.

## Materials and Methods

### Ethics Statement

We have ethical approval to work with human *post-mortem* samples (Supplementary Tables 2, 3) from the regional ethical review board of Stockholm, Sweden (EPN Dnr2012/111-31/1; 2012/2091-32). Fresh frozen tissue was obtained through the Netherlands Brain Bank (NBB). The work with human tissues was carried out according to the Code of Ethics of the World Medical Association (Declaration of Helsinki).

### Tissue Sectioning and Laser Capture

Sample preparation prior LCM-seq was carried out as follows. Frozen midbrain tissues (controls and PD), of rostral to intermediate midbrain level (Damier et al., 1999b), obtained from the brain banks were attached to chucks using pre-cooled OCT embedding medium (Histolab). 10 μm-thick coronal sections were acquired in a cryostat at −20°C and placed onto precooled-PEN membrane glass slides (Zeiss). For RNAscope experiments (control tissue), sections were cut at 12 μm-thickness and attached to Superfrost^®^ Plus slides (Thermo Scientific). The slides with sections were kept at −20°C during the sectioning and subsequently stored at −80°C until further processed. The laser capture procedure followed by sequencing library preparation (LCM-seq) was carried out as described (Nichterwitz et al., 2016, 2018). Dopamine neurons were selected based on their location and presence of neuromelanin (Supplementary Figure 2). To limit inclusion of different subpopulations within VTA and SNc, as these show different degrees of vulnerability to PD (Damier et al., 1999b), we tried to dissect cells in a precise and consistent manner. For VTA neurons, we stayed close to the midline. For SNc neurons we followed the road map of vulnerable regions within the SNc according to Damier et al. (1999b), to isolate the most susceptible neurons located in the ventral tier, which recently were shown to have a distinct transcriptional profile from the more resilient dorsal tier (Monzon-Sandoval et al., 2020).

### Mapping and Gene Expression Quantification

**FIGURE 1:**
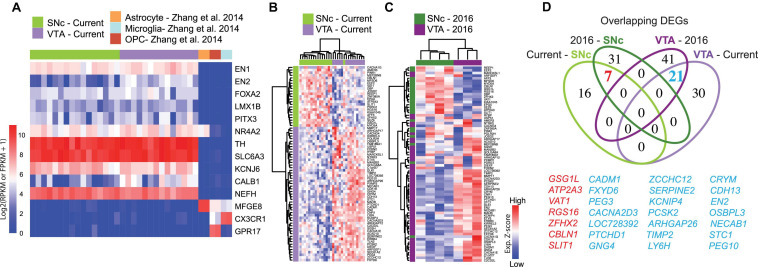
Gene signature of human adult midbrain dopamine neurons using the spatial sequencing method LCM-seq. **(A)** The high sample quality for the 18 male subjects profiled in this study was confirmed by strong expression of the midbrain dopamine neuron markers *EN1/2*, *FOXA2*, *LMX1B*, *PITX3*, *NR4A2*, *TH*, and *SLC6A3* (DAT), the pan-neuronal marker neurofilament (*NEFH*), and the lack of astrocyte, microglia or oligodendrocyte precursor marker (Yu et al., 2007) contamination. **(B)** Hierarchical clustering analysis of samples from the current study using the 74 DEGs identified by DESeq2. **(C)** The 74 DEGs also separated SNc and VTA samples from Nichterwitz et al. (2018) (3 female subjects). **(D)** Venn-diagram showing the relatively low degree of overlap between DEGs in the cohorts of different sizes (see also Supplementary Figure 2).

Samples were sequenced using an Illumina HiSeq2000, HiSeq2500, or NovaSeq platforms (reads of 43 or 50 bp in length). The uniquely mapped reads were obtained by mapping to the human reference genome hg38/GRCh38 using STAR with default settings. The reads per kilobase of transcript per million mapped reads (RPKM) were estimated using “rpkmforgenes” (Poulin et al., 2014) to 10.88 million reads and 4.7–12.3 thousand genes expressed with RPKM > 1, all samples were included. For control subjects the correlation coefficient between any two nearest samples was above 0.7. For PD samples we verified that all samples had > 1 million reads > 4600 genes expressed with RPKM > 1. For PD samples the correlation coefficient between any two nearest samples was above 0.9. It should be noted that it has been elegantly demonstrated that shallow RNA sequencing of ca 50,000 reads/cell is sufficient for unbiased cell type classification and marker gene identification of neural subclasses (Pollen et al., 2014) and thus our sequencing depth of >1 million reads/sample should be more than sufficient to subclassify SNc and VTA dopamine neurons. For all control or PD cases having more than one replicate per group, corresponding samples were averaged before analysis so that each case had only one SNc and one VTA. We confirmed the expression of known midbrain dopamine neuron markers and the purity of each sample (Figure 1A and Supplementary Figure 4).

### Differential Expression Analyses

Differentially expressed genes were identified using the R package “DESeq2” (version: 1.16.1) (Love et al., 2014) where the cutoff for significance was an adjusted *P*-value of 0.05. Identified DEGs (from different analysis and summarized below) are shown in Supplementary Tables 4, 6–9.

### Bootstrapping Approach Coupled With DESeq2

**FIGURE 2:**
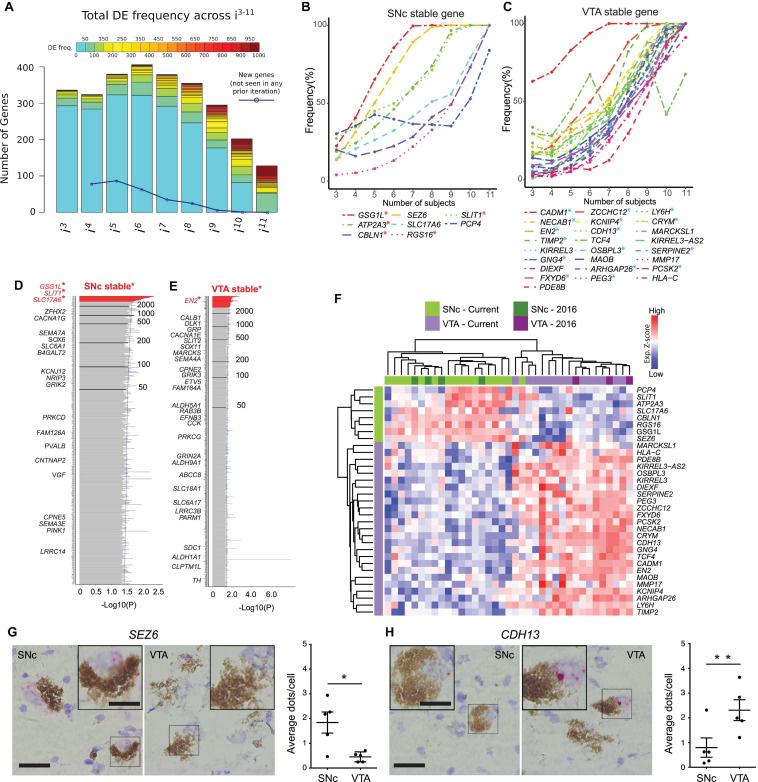
Bootstrapping analysis coupled with DESeq2 identifies SNc or VTA stable genes from a cohort of 12 human subjects. **(A)** Histogram of DEG frequency through iterative bootstrapping. *X*-axis denotes increasing size of patient pool (i^3^–i^11^ individuals) at each iteration. *Y*-axis bar height denotes the number of DE genes, while bar color denotes the frequency those genes being DE in that iteration. Blue line denotes the decreasing number of novel genes detected across successive iterations. **(B,C)** DE frequency increases at each iterative sample size increase, for SNc **(B)** or VTA **(C)** stable genes. **(D,E)** Frequency histograms (ranked by *p*-value), output of the bootstrapping approach, extend the analysis of identified SNc and VTA stable genes. Genes with the highest frequency (at the top, in red) represent SNc **(D)** and VTA **(E)** stable genes. **(F)** Hierarchical clustering analysis using the 33 stable genes, faithfully segregates both SNc and VTA samples. **(G,H)** RNAscope staining and quantification for the stable genes SEZ6 (**G**, *p* = 0.025) and CDH13 (**H**, *p* = 0.005) enriched in the SNc and VTA, respectively (*n* = 5 subjects, data represented as mean ± SEM, Paired *t*-test). Scale bars 30 μm (15 μm for insets). ^∗^*p* < 0.025 and ^∗∗^*p* < 0.005.

To counteract the variability among human subjects and identify the most reliable DEGs between SNc and VTA neurons across datasets we developed a bootstrapping approach coupled with DESeq2 (Figure 2A and Supplementary Figure 3A). The stable genes output of this analysis is correlated with the sample size and give an unbiased estimation of the number of individuals required to consistently distinguish these closely related subpopulations. Importantly this computational tool can be used for the comparison of any other two groups.

In detail:

(1) Define ¨N¨ and ¨M¨ as the number of samples in Groups 1 and 2, respectively. Choose ¨I¨ as a reference representing a given number of samples from ¨N¨ and ¨M¨.
(2) Define ¨i ¨ as the number of randomly selected samples from Groups 1 and 2, where i ∈{3, 4, 5, …, ¨I-1¨}. In the human dataset, as we have 12 paired samples, the I ∈{3, 4, 5, 6, 7, 8, 9, 10, 11}.
(3) Pool ¨i¨ samples (temporary considered a ¨new data set¨) and calculate DEGs with DESeq2.
(4) Repeat steps (2) and (3) for ¨j¨ times (set to 1,000 times in this study).
(5) For every round of random selection and DESeq2, save the full list of DEGs, compute and rank their frequency.
(6) Set a threshold (30% ratio in this study) to consider DEGs with higher frequency as stable genes.

Reliable genes appear when frequencies are above: Total times of bootstrapping × ratio (300 in this study). A stringent, but fair ratio can be defined by comparing the percentage of identified stable genes overlapping with the top (most significant) 10%, 20%, 30%, …, DEGs identified by DESeq2 alone.

### Bootstrapping Approach Applied to Human Samples

To reliable identify DEGs between human SNc and VTA samples, while minimizing subject variability, we selected 12 control individuals (66% of the dataset, 12 out of 18 individuals) where both neuronal populations were available and sequenced. Hence, the number of randomly selected samples (¨n¨ and ¨m¨ from ¨i¨ individuals) was from three to 11 and the algorithm repeated 1000 times (Supplementary Figures 3A,B).

### Bootstrapping Applied to Mouse Single Cells

For this adult mouse dataset (La Manno et al., 2016) we defined the groups SNc (*N* = 73 cells) and VTA (*M* = 170 cells comprising VTA1, VTA2, VTA3, and VTA4). To compensate the unbalance in cell number and adjust dataset representation compared to the human analysis (66%), we first randomly collected a subset of 73 VTA cells, pairing both SNc and VTA. Similarly, the number of randomly selected samples was 20, 25, 30, …, 70 and the algorithm repeated again 1,000 times.

### STRING Network Based on DE Genes Between SNc and VTA

Based on the DE genes between SNc and VTA (Supplementary Table 6), two STRING networks were created separately. The MLC clustering grouped the network into sub-networks with default parameters and marked as the dash line.

### Data Visualization

Data visualization was achieved using Principal Component Analysis (PCA) and Hierarchical Clustering (H-cluster). PCA was calculated with the function “prcomp” in R with default parameters. Then samples are projected onto the first two dimensions, PC1 and PC2. For H-cluster we used the R function “pheatmap” (version 1.0.12) with the clustering method of “ward.D2.”

### RNAscope Staining of Human Tissues

RNAscope (Wang et al., 2012) was used to verify the expression (in control tissues) of one SNc marker (*SEZ6*) and one VTA-preferential gene (*CDH13*) based on the sequencing data. In brief, midbrain sections of human fresh frozen tissue (Supplementary Table 3) were quickly thawed and fixed with fresh PFA (4% in PBS) for 1 h at 4°C. The RNAscope 2.5 HD Assay—RED Kit (Cat. 322360) was used using manufacturer recommendations. To evaluate the procedure in the midbrain tissue (Supplementary Figure 3G), we first tested a negative control probe against a bacterial gene (Cat. 310043, *dapB*-C1) and a positive control probe against tyrosine hydroxylase (Cat. 441651, *TH*-C1) (Supplementary Figure 3G). Once we set up the assay, midbrain sections were stained with *SEZ6* (Cat. 411351-C1) or *CDH13* probes (Cat. 470011-C1). Slides were counterstained with fresh 50% Gill Solution (Cat. GSH132-1L, Sigma-Aldrich) for 2 min, washed in water and dried for 15 min at 60°C before mounting with Pertex (Cat. 00811, Histolab). For every sample (*n* = 5), we imaged 5–6 random fields within the SNc and VTA regions. On average 194.25 ± 43.02 cells were imaged per region and staining. Pictures were made at 40X magnification using the bright-field of a Leica microscope (DM6000/CTR6500 and DFC310 FX camera). After randomization and coding of all the images, the number of dots within melanised cells (dopamine neurons) were counted using ImageJ (version 1.48) and later the average number of dots per cells determined for each region. Cells were classified as having either 0, 1, 2, 3, 4, 5 dots/cell. If cells had >5 dots they were classified within the 5 dots/cell category.

Investigators performing the quantification were blinded to the sample, target region (SNc and VTA) and probe staining.

### Statistical Analysis

For this study, statistical analyses were performed using ¨R¨. For the RNAscope analysis a paired *t*-test (Prism 6, Version 6.0f) was used to compare the mean average dots per cell (for *SEZ6* or *CDH13* staining) between the SNc and VTA. Where applicable, individual statistical tests are detailed in the figure legends where significance is marked by *P* < 0.05. The number of subjects/cells used for each experiment is listed in the figure or figure legends. Results are expressed as mean ± SD or SEM as specified in the figure legend.

### Data Access

All raw and processed sequencing data generated in this study have been submitted to the NCBI Gene Expression Omnibus^1^ (GEO) under accession number GSE114918. Human samples re-analyzed from the Nichterwitz study (Nichterwitz et al., 2016). ArrayExpress (E-MEXP-1416) (Cantuti-Castelvetri et al., 2007) or raw data received from Dr. Kai C. Sonntag (Simunovic et al., 2009).

## Results

### Published SNc and VTA Transcriptional Profiles Display Considerable Discrepancies

To understand the molecular underpinnings of the differential vulnerability among dopamine neurons, we compared previously published transcriptome studies of mouse and rat VTA and SNc dopamine neurons, using the list of markers reported as significantly up- or down-regulated (Grimm et al., 2004; Chung et al., 2005; Greene et al., 2005; Bifsha et al., 2014). This analysis revealed that a surprisingly low fraction of DEGs were common across data sets (Supplementary Figures 1A,B and Supplementary Tables 1, 4). Comparing across species with our previously published small data set on human SNc and VTA (Nichterwitz et al., 2016), only two genes, *SOX6* and *CALB1*, overlapped within SNc and VTA gene lists, respectively (Supplementary Figure 1C). These discrepancies highlight the urgent need to identify reproducible marker profiles for VTA and SNc dopamine neurons.

### LCM-Seq of a Large Human Cohort Identifies Markers Specific to SNc or VTA Dopamine Neurons and Suggests That Sample Size Impacts Identification of DEGs

To identify robust and specific human dopamine neuron subpopulation markers, we isolated individual VTA and SNc neurons, visualized by Histogene staining (Nichterwitz et al., 2016, 2018), from *post-mortem* tissues from 18 adult individuals by LCM (Supplementary Figures 2A–G and Supplementary Table 2) and conducted polyA-based RNA sequencing. This study represents the largest human data set profiling of SNc and VTA dopamine neurons to date. The quality of human fresh frozen tissues used may vary as a consequence of *post-mortem* interval (PMI), sample handling and preservation. Therefore, prior to conducting differential gene expression analysis we performed extensive quality control analysis to rule out undesired influences from sample processing (Supplementary Table 5). Randomly selected samples that exhibited different PMIs for VTA or SNc neurons displayed comparable cDNA quality (Supplementary Figures 2H,I). Furthermore, while the total number of reads varied between individual samples, such variability was similarly distributed between SNc and VTA samples (Supplementary Figure 2J). The number of detected genes did not correlate with either the age of the donor (Supplementary Figure 2L), the PMI (Supplementary Figure 2M) or the total number of reads (Supplementary Figure 2K). Only the number of collected cells per sample modestly impacted gene detection (*P* = 0.515) (Supplementary Figure 2N). However, neither the number of collected cells nor the number of detected genes were significantly different between SNc and VTA neuron groups (Supplementary Figures 2O,P) and thus should not affect DEG identification. Finally, we observed that all samples strongly expressed the dopamine neuron markers *EN1/2*, *FOXA2*, *LMX1B*, *PITX3*, *NR4A2*, *TH*, and *SLC6A3* (DAT), and the general neuronal marker NEFH, while they lack glial markers *MFGE8*, *CX3CR1*, or *GPR17.* This clearly demonstrates the selective enrichment of dopamine neurons using the LCM-seq methodology (Figure 1A). *KCNJ6* (GIRK2) and *CALB1*, two genes often used to distinguish between SNc or VTA dopamine neurons, were also expressed (Figure 1A), but could not, on their own, accurately differentiate our samples (Supplementary Figure 2Q).

Differential expression analysis, considering these 18 individuals, identified 74 DEGs (Supplementary Table 6), which resolved SNc from VTA neurons (Figure 1B). These genes also distinguished SNc and VTA samples in our previous small human cohort (*N* = 3) (Nichterwitz et al., 2016; Figure 1C). However, relatively few DEGs overlapped with the current large cohort (*N* = 18), even though the same experimental method was used. In fact, only seven SNc and 21 VTA DEGs overlapped across the current and the previous cohorts (Figure 1D). Notably, the 100 DEGs identified in the small cohort (*N* = 3) (Nichterwitz et al., 2016; Supplementary Figure 2R), failed to distinguish SNc and VTA in the current larger cohort of 18 subjects (Supplementary Figure 2S and Supplementary Table 7). This suggests that small sample size prevents confident identification of DEGs.

### Bootstrapping Coupled With DESeq2 Identifies Stable DEGs Unique to Human SNc or VTA

To evaluate how sample size may affect DEG detection, we used a bootstrapping algorithm in combination with DESeq2. To reduce the biological variability, we considered only those subjects for which both SNc and VTA samples were available (12/18 subjects, 24 samples in total). To begin with this approach, a subset of three subjects were randomly chosen from the pool of total 12 subjects. Differential expression analysis was then performed between the SNc and VTA samples of these subjects (DESeq2), and DEGs were selected with adj *P* < 0.05. This random sampling of three subjects, followed by DESeq2 analysis, was performed a total of 1,000 times, and the DE frequency over these 1,000 comparisons was recorded for this iteration (*i* = 3 subjects). Subsequently, this process was repeated using subsets of four subjects, then five, up to a maximum of 11 of the 12 subjects. For each subset size (i^3^–i^11^)the DEG frequency was calculated by the 1,000x comparisons of that iteration (Supplementary Figure 3A). By considering all DEGs in an iteration we were able to detect hundreds of genes, where on average only e.g., 13 DEGs were detected in i^3^ (Supplementary Figure 3B). We found that the number of DEGs decreased with increased subset size, while the detection frequency increased. More importantly, the number of new DEGs detected also decreased with increasing sample size (Figure 2A). Interestingly, we found that in our cohort eight subjects were required to saturate the DEG detection, as few new genes were identified when considering additional subjects in subsequent iterations (Figure 2A, blue line). We identified eight stable genes for the SNc (Figure 2B) and 25 stable genes for the VTA (Figure 2C). Five of the SNc stable genes (labeled in red**^∗^,** see Figure 1D: *GSG1L*, *ATP2A3*, *CBLN1*, *RGS16*, and *SLIT1*) and 16 of the VTA stable genes (in blue**^∗^**, see Figure 1D: *CADM1*, *NECAB1*, *EN2*, *TIMP2*, *GNG4*, *FXYD6*, *ZCCHC12*, *KCNIP4*, *CDH13*, *OSBPL3*, *ARHGAP26*, *PEG3*, *LYH6*, *CRYM*, *SERPINE2*, and *PCSK2*) were among the aforementioned seven and 21 overlapping DEGs, that we identified across the two studies.

We then summed DEGs across i^3^–i^11^ (9,000 comparisons in total), separated genes into SNc or VTA enriched lists, and ranked the lists from most to least frequently DEs (Figures 2D,E; Supplementary Figures 3C,D and Supplementary Table 8). Highly ranked genes on these two lists included multiple known SNc and VTA markers in human (e.g., *GSG1L*, *SLIT1*, *ATP2A3*, *CADM1*, *CRYM*, and *TCF4*) (Schultzberg et al., 1984; Cantuti-Castelvetri et al., 2007; La Manno et al., 2016; Nichterwitz et al., 2016). To identify the most reliable DEGs, we designated genes that were detected more than 3,000 times (out of 9,000) as “stable genes.” This stringent cutoff was chosen since the resulting SNc and VTA lists would then contain at least one stable gene that could be identified during the first iteration (where three individuals were used as the sample size). Frequency histograms (ranked by *p*-value) as the output of the overall bootstrapping approach showed genes with the 30% threshold (at the top, in red) represent SNc and VTA stable genes (Supplementary Figure 3C). We also compared this stable gene list with the outcome of DESeq2 analysis alone, when applied to the same 12 subjects (Supplementary Figures 3D,E). All eight SNc and 25 VTA markers perfectly overlapped with the DEGs from DESeq2 alone using an adjusted *P* < 0.05 (Supplementary Figure 3D) or a more stringent significance (adj. *P* < 0.01, Supplementary Figure 3E). The expression of SNc stable genes was confirmed in two independent human microarray datasets which only analyzed SNc neurons (Supplementary Figure 3F; Cantuti-Castelvetri et al., 2007; Simunovic et al., 2009). Importantly, the stable genes faithfully classified SNc and VTA from 21 individuals (Figure 2F), namely all 18 male individuals from our current dataset and the three female samples investigated previously (Nichterwitz et al., 2016). Moreover, using RNA scope we confirmed the subpopulation-specific expression pattern of the identified SNc stable gene *SEZ6* (Figure 2G) and the VTA stable gene *CDH13* (Figure 2H) in human *post-mortem* tissues, further ratifying our LCM-seq data and the bootstrapping approach.

In conclusion, we have identified 33 markers that correctly classify samples as either SNc or VTA, and that remain robust to individual subject variability. Notably, these genes were stably differentially expressed only when at least eight subjects were included in the bootstrapping strategy (Figures 2B,C). Thus, we have defined the minimal sample size required to distinguish SNc and VTA subpopulations in human subjects using LCM-seq and show that DEG identification below this number is unreliable. Depending on the variability among samples within a particular cohort this number could vary and should thus first be defined for each new cohort. The variability in DEGs identified between SNc and VTA dopamine neurons among previous studies could in part be explained by their use of too small cohorts.

To further validate our bootstrapping approach, we applied it to a published, postnatal, mouse single-cell dataset profiling midbrain dopamine neurons (La Manno et al., 2016; Supplementary Figure 4A, raw data analyzed here). Single cells were initially assessed for expression of known dopamine neuron markers and the absence of contaminating glia or oligodendrocyte markers (Supplementary Figure 4B; Zhang et al., 2014). All available SNc dopamine neurons (73 in total) and 73 randomly selected VTA dopamine neurons were then subjected to aforementioned bootstrapping followed by DESeq2, through which we identified 36 SNc-enriched transcripts and 53 VTA-enriched transcripts (Supplementary Figures 4C,D and Supplementary Table 9). These stable gene sets for SNc and VTA included novel genes in addition to previously reported markers (Grimm et al., 2004; Chung et al., 2005; Greene et al., 2005; Bifsha et al., 2014; Poulin et al., 2014; La Manno et al., 2016; Nichterwitz et al., 2016).

Importantly, these 89 stable genes, identified through our bootstrapping approach, effectively classified the single cells into the correct population, SNc or VTA (Supplementary Figure 4E) and the specific expression patterns in either SNc or VTA was corroborated in the adult mouse using Allen *in situ* images as exemplified in Supplementary Figure 4F. Specific expression patterns within either SNc or VTA was confirmed using Allen Brain Atlas, see examples of *Serpine2*, *Zcchc1*, and *Cdh13* in coronal midbrain sections (Supplementary Figure 4F). Finally, we wanted to see how the stable DEGs would overlap with DEGs identified through DEseq2 alone. We first plotted the number of DEGs identified through DESeq2 as a function of the adjusted *P*-value with an evident and expected decrease in the number of identified DEGs between SNc and VTA with stricter *P*-values (Supplementary Figure 4G). We then plotted the stable SNc and VTA DEGs and the DEGs identified through DESeq2 alone at an adjusted *P* = 0.05. The resulting Venn diagram shows that the majority of stable DEGs identified through our bootstrapping approach were also included when DESeq2 alone was used, with 50 out of 53 stable VTA DEGs and 25 out of 36 stable SNc DEGs being identified (Supplementary Figure 4H).

In conclusion, our bootstrapping strategy could be reliably applied to another larger data set and used to define stable SNc and VTA markers between two highly similar populations.

### STRING Analysis Identifies Novel Networks for Human SNc and VTA Dopamine Neurons and Highlights Cellular Functions That Uniquely Define Each Subpopulation

**FIGURE 3:**
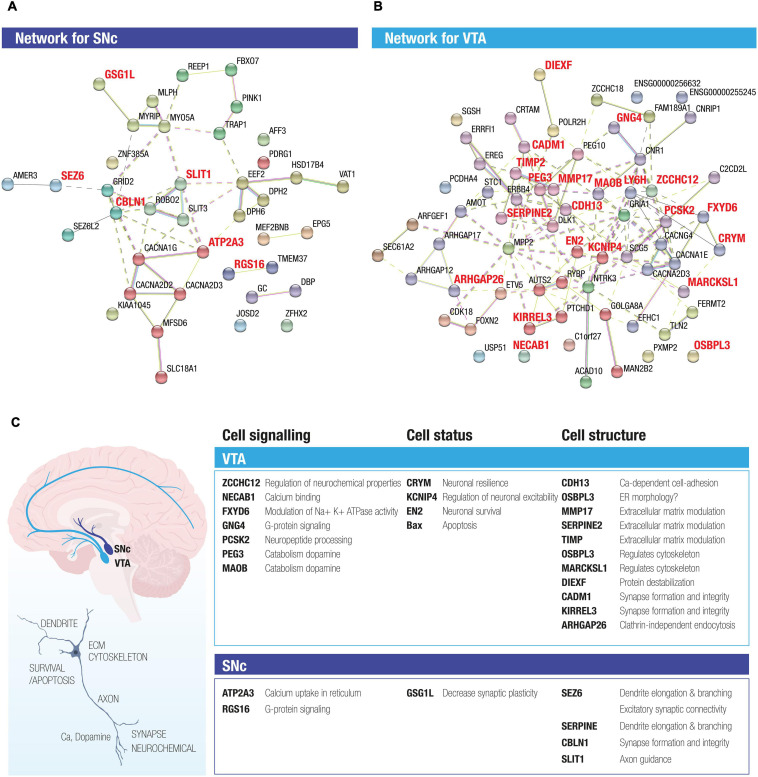
STRING analysis of DEGs between SNc and VTA identifies novel networks for the two midbrain dopamine neuron subpopulations. The two STRING networks are based on 23 DE genes highly expressed in SNc **(A)** and 51 DE genes highly expressed in VTA **(B)**. Different color edges represent the interactions by curated databases, experiment or prediction. The nodes of the two networks are grouped using dash line by using MCL clustering, in which the nodes with solid edges are from sub-networks. **(C)** The activity of the DEGs present in the SNc and VTA networks highlight both possible beneficial functions as well as functions that could render neurons susceptible, which were predominant in the resilient versus vulnerable dopamine neuron subpopulations. Proposed enriched functions include e.g., neuronal survival, regulation of mitochondrial stability, catabolism of dopamine, regulation of resting membrane potential, extracellular matrix modulation and regulation of cytoskeleton and synapse integrity, G protein signaling and calcium uptake.

To explore potential interactions among the stable DEGs in human SNc and VTA dopamine neurons, we conducted STRING analysis. For this purpose, we used the 74 DEGs, including the stable DEGs, retrieved by comparing SNc and VTA, as the input (Supplementary Table 5). The two STRING networks shown are thus based on 23 genes with preferential expression in SNc (Figure 3A) and 51 genes with predominant expression in VTA (Figure 3B). The interactions between genes are shown through different color edges and were curated from databases, experiment or prediction. The nodes of the two networks are grouped using dashed lines by using MCL (Markov Clustering), in which the nodes with solid edges are from sub-networks. The interactions of the DEGs present in the VTA network highlight possible beneficial functions that are predominant in this resilient dopamine neuron subpopulation, including induction of survival genes, regulation of mitochondrial stability, catabolism of dopamine, regulation of resting membrane potential, extracellular matrix modulation, and regulation of cytoskeleton and synapse integrity (Figure 3C). The identified enriched gene networks give clues to networks that underlie the subpopulations unique functions and likely their differences in susceptibility.

### The Stable DEGs Identified in Control Tissues Also Define SNc and VTA Subpopulations in PD

**FIGURE 4:**
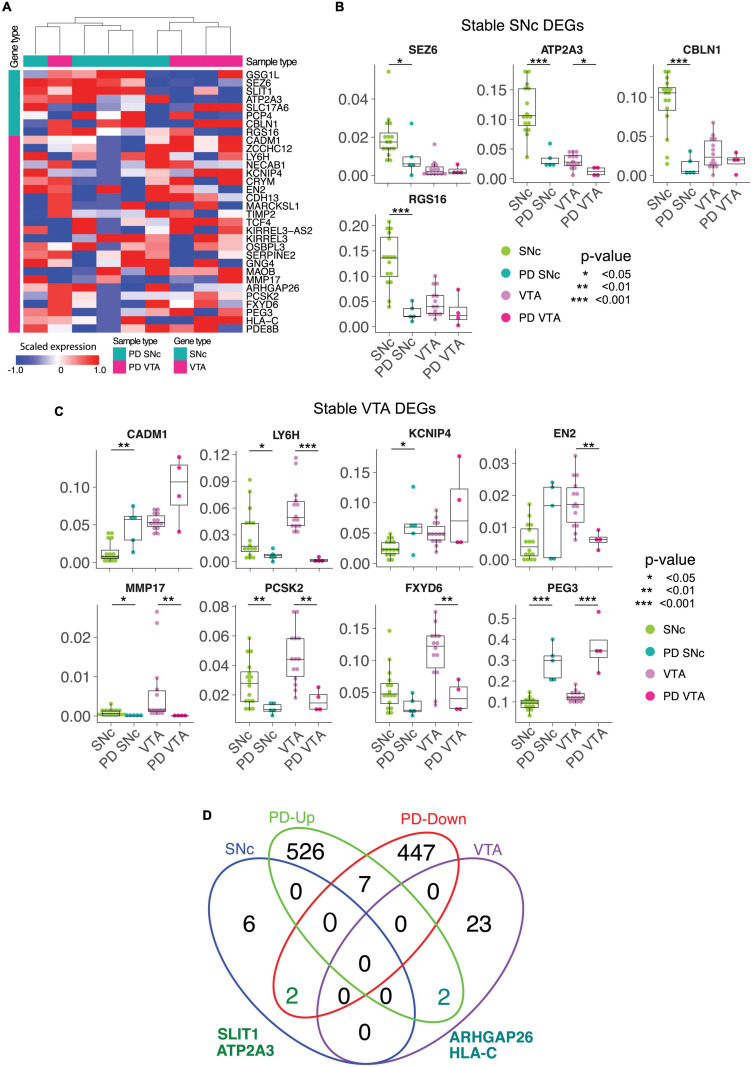
The stable DEGs identified in control tissue separates SNc and VTA from Parkinson’s disease patients. **(A)** The stable DE genes hierarchically separate SNc and VTA from both normal and Parkinson’s disease (PD) patients. **(B,C)** The differential expressions of stable DE genes between normal and PD patients in SNc and VTA are shown. **(D)** Venn diagram finds two common genes, SLIT1 and ATP2A3, between SNc stable and down-regulated genes in PD SNc. ^∗^*p* < 0.05, ^∗∗^*p* < 0.05, ^∗∗∗^*p* < 0.05.

We conducted LCM-seq on PD patient tissues to understand if the stable DEGs identified in control tissues would still define SNc and VTA dopamine neurons in end-stage disease. Hierarchical clustering of SNc and VTA PD samples using the stable DEGs separated the majority of samples into the expected subtypes, only one sample out of each group misclassified using this approach. This indicates that the stable genes still define the uniqueness of these two dopamine neuron subpopulations in disease (Figure 4A). Analysis of individual DEGs showed that *SEZ6*, *ATP2A3*, *CBLN1*, and *RGS16* maintained a preferential expression in SNc versus VTA dopamine neurons also in PD, although the expression was lower in PD than control SNc (Figure 4B). Similarly, *LY6H*, *MMP17*, *EN2*, *PCSK2*, *FXYD6*, and *PEG3*, defined the VTA subclass of dopamine neurons also in PD, but were also in general lower in PD (Figure 4C). Thus, the identified markers can be used to study the two subpopulations both in health and PD. However, while our small PD cohort indicates that the markers may be affected by the disease process we need a larger cohort to confirm and solidify findings. Therefore, we analyzed the expression of the stable DEGs in a larger cohort of PD samples where SNc dopamine neuron gene expression alone was analyzed in health and PD (Schultzberg et al., 1984). Our analysis of this larger PD cohort confirmed our finding from our smaller PD cohort that the SNc stable DEG *ATP2A3* was significantly down-regulated in disease (Figure 4D). In the larger PD cohort, *SLIT1*, another SNc stable gene, was also found to be significantly down-regulated in PD (Figure 4D), something we did not detect in our smaller PD cohort. Furthermore, two stable VTA DEGs, ARHGAP26 and HLA-C, were up-regulated in the SNc of the large PD cohort (Figure 4D). The marked decrease of *SLIT1* and *ATP2A3*, and the increase of ARHGAP26 and HLA-C, is a novel PD signature which could be further explored to evaluate neuronal resilience and vulnerability.

## Discussion

The selective vulnerability of SNc dopamine neurons to PD, and the relative resilience of VTA dopamine neurons, has encouraged the field to investigate the molecular signature of these two neuron subpopulations. When we analyzed existing data sets (Grimm et al., 2004; Chung et al., 2005; Greene et al., 2005; Bifsha et al., 2014; Poulin et al., 2014; La Manno et al., 2016; Nichterwitz et al., 2016), we identified large discrepancies in the reported SNc or VTA enriched genes across different studies. This could result from multiple factors, including small sample sizes and variability between subjects, which is recognized to be a major confounding factor in human studies (Mele et al., 2015). This prompted us to conduct a large focused study on adult human midbrain dopamine neurons using LCM-seq (Nichterwitz et al., 2018). We consequently constructed a comprehensive LCM-seq dataset, isolating single SNc or VTA dopamine neurons from *post-mortem* tissues of total 18 individuals, the largest collection of human dopamine neurons, aiming to reveal robust molecular signatures to distinguish the two subpopulations.

Using an iterative bootstrapping without replacement coupled with DESeq2 (available at https://github.com/shanglicheng/BootstrappingWithoutReplacement), and a strict selection-criteria (here, a 30% threshold for stable classification) we identify 33 of the most stable DEGs. Among these, 25 of the genes define VTA identity, while eight define SNc identity, which together accurately classify LCM-seq samples from our previous (three females), and current (18 males) subject cohorts. We confirm the utility of our bootstrapping approach on a larger published mouse single cell data set and show that identified DEGs there could correctly classify SNc and VTA dopamine neurons.

Using our approach, we also identify a minimal sample size required to identify human stable genes, which for our cohort was an *N* = 8. The sample size may of course vary depending on the specifics of the cohort and the similarity of the subpopulations to be compared. However, our approach clearly demonstrates that to identify lineage-specific markers between any two highly related cellular subpopulations it is of utter importance to determine the sample size and to use a sufficiently large cohort size. Such considerations also apply to studies comparing, for example, healthy and diseased dopamine neurons that may exhibit potentially subtle pathological changes.

The identified stable DEGs highlighted that VTA and SNc dopamine neurons display differences in several important functions such as cytoskeletal regulation, extracellular matrix modulation, synapse integrity, mitochondrial stability, regulation of apoptosis, and neuronal survival. The VTA-predominant transcript *SERPINE2* (Glia-derived nexin) is a serine protease inhibitor which can promote neurite extension by inhibiting thrombin, and which appears down-regulated in Alzheimer’s disease (Choi et al., 1995). Serpine2 promotes biogenesis of secretory granule which is required for neuropeptide sorting, processing and secretion (Kim and Loh, 2006). *CDH13*, another VTA-specific transcript, encodes for adhesion protein 13, which together with other family members as *CDH9* and *CDH15* are linked to neuropsychiatric disorders (Redies et al., 2012). Cdh13 can regulate neuronal migration and also has an effect on axonal outgrowth as demonstrated in the serotonergic system (Forero et al., 2017). The VTA-predominant gene Engrailed-2 (EN2) is a transcription factor known to promote survival of dopamine neurons by inducing survival gene expression and by protecting neurons from oxidative stress and blocking mitochondrial instability (Alavian et al., 2009; Alvarez-Fischer et al., 2011; Rekaik et al., 2015). The higher level of *EN-2* in VTA compared to SNc neurons could in part explain the relative resilience of these neurons to PD. The stable DEGs we identified here may be highly relevant to induce resistance or model disease as previously attempted in rodents (Chung et al., 2005; Poulin et al., 2014). Several of the human stable genes (or related family members), e.g., *GSG1L*, *ATP2A3*, *SLC17A6*, *SLIT1,RGS16*, *KCNIP1*, *CDH13*, *TCF12*, *OSBPL1A*, *OSBPL10*, *GNG7*, *ARHGAP18*, *ARHGAP24*, *PCSK5*, *PEG3*, *HLA-DOA*, *HLA-DRA*, *HLA-DRB1*, and *PDE8B* are dysregulated in PD (Cantuti-Castelvetri et al., 2007; Bossers et al., 2009; Simunovic et al., 2009) and/or are represented in PD datasets from genome wide association studies^2^ (GWASdb SNP-Disease Associations dataset). Interestingly, mice lacking *Rgs6*, a related family member of the human SNc stable gene *RGS16*, develop specific degeneration and cell loss of SNc dopamine neurons at the age of 12 months (Bifsha et al., 2014). It remains to be investigated if *RGS16* has a similar function. Loss of the SNc stable gene *Cplx1* results in a compromised nigrostriatal pathway in knockout mice (Hook et al., 2018). Moreover, mutations in the human SNc stable gene *SEZ6* have been implicated in diseases such as Alzheimer’s (Khoonsari et al., 2016; Paracchini et al., 2018), childhood-onset schizophrenia (Ambalavanan et al., 2016), epilepsy and febrile seizures (Yamada et al., 1990; Mulley et al., 2011). CALBINDIN 1 (CALB1) is often used as a marker unique to VTA dopamine neurons. The rank of *CALB1* on the VTA list was just below the 30% frequency threshold for the “stable gene” classification (Figure 2E). However, while CALB1 is present in the majority of VTA dopamine neurons it is also present in a selection of SNc dopamine neurons (Parent et al., 1996) and thus it is not surprising that it did not make it onto the stable gene list. Notably, it may be a general marker of resilient dopamine neurons as CALB1^+^ neurons in the SNc show relative sparing in Parkinson’s disease (Yamada et al., 1990).

Analysis of stable DEG expression in PD material showed that *SEZ6*, *ATP2A3*, *CBLN1*, and *RGS16* maintained preferential expression in SNc versus VTA dopamine neurons also in disease. Similarly, *LY6H*, *MMP17*, *EN2*, *PCSK2*, *FXYD6*, and *PEG3*, defined the VTA subclass of dopamine neurons also in PD. Analysis of the stable DEGs identified in control brains in a larger cohort of PD samples where SNc dopamine neuron gene expression was analyzed demonstrated that two genes, *SLIT1* and *ATP2A3*, out of the eight stable SNc DEGs were dysregulated in PD. This could indicate that these two markers are mainly expressed in the most vulnerable SNc dopamine neurons that are no longer present in end-stage PD patient tissues. However, it is also possible that these two genes are down-regulated in general in all SNc neurons. Future single cell analysis of human dopamine neurons throughout disease progression in PD could aid in discriminating between these two possible scenarios. Nonetheless the marked down-regulation of these two markers in PD can be used to distinguish disease-afflicted from healthy SNc dopamine neurons. SLIT1 appears to block neurite extension of dopamine neurons (Lin and Isacson, 2006). The loss of *SLIT1* may be a compensatory response of remaining cells to allow for neurite growth during disease. Notably, mutant PD-causative forms of LRRK2 induce dystrophic neurites and can also decrease the number of neurites (MacLeod et al., 2006; Li et al., 2009), indicating that it would be beneficial for dopamine neurons to counteract such processes by modulating the transcriptome accordingly to promote neurite extension. Alternatively, it is possible that the SNc neurons that had high levels of *SLIT1* were lost earlier in the PD process due to their inability to modulate neurite extension. This would parallel the situation in amyotrophic lateral sclerosis where motor neurons having high levels of the growth repellant factor EPHA4 are the neurons that are unable to sprout and reconnect with muscle targets and which are consequently lost first in disease (Van Hoecke et al., 2012). It would be feasible to distinguish between these two possible scenarios using single cell RNA sequencing from *post-mortem* PD tissues from different disease stages.

The lower levels of *ATP2A3*, an ATPase which transports Ca^2+^ across membranes to the endoplasmatic reticulum to maintain a low cytoplasmic Ca^2+^ level, in PD SNc neurons, indicates a deficit in organelle function and Ca^2+^sequestration. Increased levels of cytoplasmic Ca^2+^ due to lowered ATP2A3 levels could be detrimental to cells and cause degeneration (Bezprozvanny, 2009). This data would indicate that remaining SNc neurons have dysfunctions in important cellular processes that need to be tightly regulated by Ca^2+^ levels.

The increased level of the stable VTA DEGs, *HLA-C* and *ARHGAP26*, in SNc PD dopamine neurons is very compelling. *ARHGAP26* was recently identified as a potential early, diagnostic biomarker for PD, as it was found up-regulated in the blood of PD patients (Jiang et al., 2019). ARHGAP26 is a Rho GTPase activating protein which is involved in regulating actin-cytoskeleton organization in response to interaction with the extracellular matrix, by mediating RhoA and Cdc42 activity (Taylor et al., 1999). It would be interesting to study potential structural modifications, resulting from cytoskeletal remodeling, of SNc dopamine neurons in response to PD, and possible effects on their connectome to evaluate if modulating such fundamental processes is part of a protective or detrimental response. HLA-C is a leukocyte antigen that is part of the major histocompatibility complex (MHC)-I, which presents short peptides to the immune system. It has been shown that MHC-I is induced in neurons by factors released from activated microglia, which is a prominent feature of the neuroinflammatory response seen in PD patient tissues. This neuronal MHC-I expression can trigger an antigenic response and cause dopamine neuron death through T-cell mediated cytotoxicity (Cebrian et al., 2014). Thus, an up-regulation of *HLA-A* as we see in PD SNc dopamine neurons is likely to be detrimental to the cells.

Regarding cell replacement therapies targeting PD (Alavian et al., 2009; Kriks et al., 2011; Ganat et al., 2012; Kefalopoulou et al., 2014; Kirkeby et al., 2017), there is still an urgent need to optimize the pluripotent stem cell preparations to specifically generate SNc rather than VTA neurons (Barker et al., 2017; Sonntag et al., 2018). Evaluation of the correct patterning and differentiation of pluripotent cells to midbrain dopamine neurons relies upon gene expression analysis using quantitative real time PCR (qPCR) or global transcriptome approaches such as RNA sequencing (Ganat et al., 2012; Barker et al., 2017; Nolbrant et al., 2017; Studer, 2017). Hence, accurate reference gene signatures of adult human SNc neurons are critical toward further advancements in the regenerative PD field. Our LCM-seq and computational stable gene analysis can therefore serve as a reference describing the transcriptional profile of adult, human SNc, and VTA neurons. This will greatly facilitate dopamine neuron replacement efforts, in addition to disease modeling studies using dopamine neurons derived from patient-specific pluripotent cells (Miller et al., 2013; Vera et al., 2016).

In summary, using LCM-seq to isolate individual dopamine neurons from SNc and VTA followed by a bootstrapping approach coupled with DESeq2 analysis, we have identified reliable SNc and VTA dopamine neuron markers in human and show that these are relevant also in PD patient tissues. We reveal the smallest human cohort size required to detect such stable DEGs, informing future study designs targeting highly related cellular populations and highlighting that DEGs detected below this cohort size are unreliable. We also demonstrate that a few SNc markers are modulated in PD and could be highly relevant as biomarkers of disease and to understand disease mechanisms further. This human transcriptomic data set, derived from individually isolated dopamine neurons, will thus help further our understanding and modeling of selective neuronal vulnerability and resilience, and serve as a reference for derivation of authentic SNc or VTA dopamine neurons from stem cells.

## Data Availability Statement

The datasets presented in this study can be found in online repositories. The names of the repository/repositories and accession number(s) can be found in the article/Supplementary Material.

## Author Contributions

EH, QD, and JA: conceptualization and funding acquisition. JA, MC, SC, MW, NK, QD, and EH: methodology, investigation, writing—review, and editing. SC, JA, MW, and NK: software, formal analysis, and visualization. JA, SC, QD, and EH: writing-original draft. EH and QD: supervision and project administration. All authors contributed to the article and approved the submitted version.

## Conflict of Interest

The authors declare that the research was conducted in the absence of any commercial or financial relationships that could be construed as a potential conflict of interest.

## Footnotes

1 https://www.ncbi.nlm.nih.gov/geo/

2 http://amp.pharm.mssm.edu

## References

[B1] Alavian K. N. Sgado P. Alberi L. Subramaniam S. Simon H. H. (2009). Elevated P75NTR expression causes death of engrailed-deficient midbrain dopaminergic neurons by Erk1/2 suppression. *Neural Dev.* 4:11. 10.1186/1749-8104-4-11 19291307 PMC2667502

[B2] Alvarez-Fischer D. Fuchs J. Castagner F. Stettler O. Massiani-Beaudoin O. Moya K. L. (2011). Engrailed protects mouse midbrain dopaminergic neurons against mitochondrial complex I insults. *Nat. Neurosci.* 14 1260–1266. 10.1038/nn.2916 21892157

[B3] Ambalavanan A. Girard S. L. Ahn K. Zhou S. Dionne-Laporte A. Spiegelmann D. (2016). De novo variants in sporadic cases of childhood onset schizophrenia. *Eur. J. Hum. Genet.* 24 944–948. 10.1038/ejhg.2015.218 26508570 PMC4867457

[B4] Barker R. A. Parmar M. Studer L. Takahashi J. (2017). Human trials of stem cell-derived dopamine neurons for Parkinson’s disease: dawn of a new era. *Cell Stem Cell* 21 569–573. 10.1016/j.stem.2017.09.014 29100010

[B5] Bezprozvanny I. (2009). Calcium signalling and neurodegenerative diseases. *Trends Mol. Med.* 15 89–100. 19230774 10.1016/j.molmed.2009.01.001 PMC3226745

[B6] Bifsha P. Yang J. Fisher R. A. Drouin J. (2014). Rgs6 is required for adult maintenance of dopaminergic neurons in the ventral substantia nigra. *PLoS Genet.* 10:e1004863. 10.1371/journal.pgen.1004863 25501001 PMC4263397

[B7] Bossers K. Meerhoff G. Balesar R. van Dongen J. W. Kruse C. G. Swaab D. F. (2009). Analysis of gene expression in Parkinson’s disease: possible involvement of neurotrophic support and axon guidance in dopaminergic cell death. *Brain Pathol.* 19 91–107. 10.1111/j.1750-3639.2008.00171.x 18462474 PMC8094761

[B8] Cantuti-Castelvetri I. Keller-McGandy C. Bouzou B. Asteris G. Clark T. W. Frosch M. P. (2007). Effects of gender on nigral gene expression and parkinson disease. *Neurobiol. Dis.* 26 606–614. 10.1016/j.nbd.2007.02.009 17412603 PMC2435483

[B9] Cebrian C. Zucca F. A. Mauri P. Steinbeck J. A. Studer L. Scherzer C. R. (2014). MHC-I expresson reders catecholainergic neurons susceptible to T-cell-mediated degeneration. *Nat. Commun.* 5:3633. 10.1038/ncomms4633 PMC4024461 24736453

[B10] Choi B. H. Kim R. C. Vaughan P. J. Lau A. Van Nostrand W. E. Cotman C. W. (1995). Decreases in protease nexins in Alzheimer’s disease brain. *Neurobiol. Aging* 16 557–562. 10.1016/0197-4580(95)00060-r 8544905

[B11] Chung C. Y. Seo H. Sonntag K. C. Brooks A. Lin L. Isacson O. (2005). Cell type-specific gene expression of midbrain dopaminergic neurons reveals molecules involved in their vulnerability and protection. *Hum. Mol. Genet.* 14 1709–1725. 10.1093/hmg/ddi178 15888489 PMC2674782

[B12] Dahlstroem A. Fuxe K. (1964). Evidence for the existence of monoamine-containing neurons in the central nervous system. I. Demonstration of monoamines in the cell bodies of brain stem neurons. *Acta Physiol. Scand. Suppl*. SUPPL 232, 231–255. 14229500

[B13] Damier P. Hirsch E. C. Agid Y. Graybiel A. M. (1999a). The substantia nigra of the human brain. I. Nigrosomes and the nigral matrix, a compartmental organization based on calbindin D(28K) immunohistochemistry. *Brain* 122(Pt 8) 1421–1436. 10430829 10.1093/brain/122.8.1421

[B14] Damier P. Hirsch E. C. Agid Y. Graybiel A. M. (1999b). The substantia nigra of the human brain. II. Patterns of loss of dopamine-containing neurons in Parkinson’s disease. *Brain* 122(Pt 8) 1437–1448. 10430830 10.1093/brain/122.8.1437

[B15] Di Salvio M. Di Giovannantonio L. G. Omodei D. Acampora D. Simeone A. (2010). Otx2 expression is restricted to dopaminergic neurons of the ventral tegmental area in the adult brain. *Int. J. Dev. Biol.* 54 939–945. 10.1387/ijdb.092974ms 19924631

[B16] Forero A. Rivero O. Waldchen S. Ku H. P. Kiser D. P. Gartner Y. (2017). Cadherin-13 deficiency increases dorsal raphe 5-HT neuron density and prefrontal cortex innervation in the mouse brain. *Front. Cell. Neurosci.* 11:307. 10.3389/fncel.2017.00307 29018333 PMC5623013

[B17] Ganat Y. M. Calder E. L. Kriks S. Nelander J. Tu E. Y. Jia F. (2012). Identification of embryonic stem cell-derived midbrain dopaminergic neurons for engraftment. *J. Clin. Invest.* 122 2928–2939. 10.1172/jci58767 22751106 PMC3408729

[B18] Greene J. G. Dingledine R. Greenamyre J. T. (2005). Gene expression profiling of rat midbrain dopamine neurons: implications for selective vulnerability in parkinsonism. *Neurobiol. Dis.* 18 19–31. 10.1016/j.nbd.2004.10.003 15649693

[B19] Grimm J. Mueller A. Hefti F. Rosenthal A. (2004). Molecular basis for catecholaminergic neuron diversity. *Proc. Natl. Acad. Sci. U.S.A.* 101 13891–13896. 10.1073/pnas.0405340101 15353588 PMC518849

[B20] Haque N. S. LeBlanc C. J. Isacson O. (1997). Differential dissection of the rat E16 ventral mesencephalon and survival and reinnervation of the 6-OHDA-lesioned striatum by a subset of aldehyde dehydrogenase-positive TH neurons. *Cell Transplant.* 6 239–248. 10.1016/s0963-6897(97)86921-7 9171157

[B21] Hedlund E. Perlmann T. (2009). Neuronal cell replacement in Parkinson’s disease. *J. Intern. Med.* 266 358–371. 10.1111/j.1365-2796.2009.02155.x 19765180

[B22] Hook P. W. McClymont S. A. Cannon G. H. Law W. D. Morton A. J. Goff L. A. (2018). Single-cell RNA-seq of mouse dopaminergic neurons informs candidate gene selection for sporadic Parkinson disease. *Am. J. Hum. Genet.* 102 427–446. 10.1016/j.ajhg.2018.02.001 29499164 PMC5985341

[B23] Jiang F. Wu Q. Sun S. Bi G. Guo L. (2019). Identification of potential diagnostic biomarkers for Parkinson’s disease. *FEBS Open Bio* 9 1460–1468. 10.1002/2211-5463.12687 31199560 PMC6668373

[B24] Kefalopoulou Z. Politis M. Piccini P. Mencacci N. Bhatia K. Jahanshahi M. (2014). Long-term clinical outcome of fetal cell transplantation for Parkinson disease: two case reports. *JAMA Neurol.* 71 83–87. 10.1001/jamaneurol.2013.4749 24217017 PMC4235249

[B25] Khoonsari P. E. Haggmark A. Lonnberg M. Mikus M. Kilander L. Lannfelt L. (2016). Analysis of the cerebrospinal fluid proteome in Alzheimer’s disease. *PLoS One* 11:e0150672. 10.1371/journal.pone.0150672 26950848 PMC4780771

[B26] Kim T. Loh Y. P. (2006). Protease nexin-1 promotes secretory granule biogenesis by preventing granule protein degradation. *Mol. Biol. Cell* 17 789–798. 10.1091/mbc.e05-08-0755 16319172 PMC1356589

[B27] Kirkeby A. Nolbrant S. Tiklova K. Heuer A. Kee N. Cardoso T. (2017). Predictive markers guide differentiation to improve graft outcome in clinical translation of hESC-based therapy for Parkinson’s disease. *Cell Stem Cell* 20 135–148. 10.1016/j.stem.2016.09.004 28094017 PMC5222722

[B28] Kriks S. Shim J. W. Piao J. Ganat Y. M. Wakeman D. R. Xie Z. (2011). Dopamine neurons derived from human ES cells efficiently engraft in animal models of Parkinson’s disease. *Nature* 480 547–551. 10.1038/nature10648 22056989 PMC3245796

[B29] La Manno G. Gyllborg D. Codeluppi S. Nishimura K. Salto C. Zeisel A. (2016). Molecular diversity of midbrain development in mouse, human, and stem cells. *Cell* 167 566–580.e19. 27716510 10.1016/j.cell.2016.09.027 PMC5055122

[B30] Li Y. Liu W. Oo T. F. Wang L. Tang Y. Jackson-Lewis V. (2009). Mutant LRRK2(R1441G) BAC transgenic mice recapitulate cardinal features of Parkinson’s disease. *Nat. Neurosci.* 12 826–828. 10.1038/nn.2349 19503083 PMC2845930

[B31] Lin L. Isacson O. (2006). Axonal growth regulation of fetal and embryonic stem cell-derived dopaminergic neurons by Netrin-1 and Slits. *Stem Cells* 24 2504–2513. 10.1634/stemcells.2006-0119 16840550 PMC2613222

[B32] Love M. I. Huber W. Anders S. (2014). Moderated estimation of fold change and dispersion for RNA-seq data with DESeq2. *Genome Biol.* 15:550. 10.1186/s13059-014-0550-8 PMC4302049 25516281

[B33] MacLeod D. Dowman J. Hammond R. Leete T. Inoue K. Abeliovich A. (2006). The familial Parkinsonism gene LRRK2 regulates neurite process morphology. *Neuron* 52 587–593. 10.1016/j.neuron.2006.10.008 17114044

[B34] Mele M. Ferreira P. G. Reverter F. DeLuca D. S. Monlong J. Sammeth M. (2015). Human genomics. The human transcriptome across tissues and individuals. *Science* 348 660–665. 25954002 10.1126/science.aaa0355 PMC4547472

[B35] Miller J. D. Ganat Y. M. Kishinevsky S. Bowman R. L. Liu B. Tu E. Y. (2013). Human iPSC-based modeling of late-onset disease via progerin-induced aging. *Cell Stem Cell* 13 691–705. 10.1016/j.stem.2013.11.006 24315443 PMC4153390

[B36] Monzon-Sandoval J. Poggiolini I. Ilmer T. Wade-Martins R. Webber C. Parkkinen L. (2020). Human-specific transcriptome of ventral and dorsal midbrain dopamine neurons. *Ann. Neurol.* 87 853–868. 10.1002/ana.25719 32167609 PMC8651008

[B37] Mulley J. C. Iona X. Hodgson B. Heron S. E. Berkovic S. F. Scheffer I. E. (2011). The role of seizure-related SEZ6 as a susceptibility gene in febrile seizures. *Neurol. Res. Int.* 2011:917565. 10.1155/2011/917565 PMC3139179 21785725

[B38] Nichterwitz S. Benitez J. A. Hoogstraaten R. Deng Q. Hedlund E. (2018). LCM-seq: a method for spatial transcriptomic profiling using laser capture microdissection coupled with PolyA-based RNA sequencing. *Methods Mol. Biol.* 1649 95–110. 10.1007/978-1-4939-7213-5_6 29130192

[B39] Nichterwitz S. Chen G. Aguila Benitez J. Yilmaz M. Storvall H. Cao M. (2016). Laser capture microscopy coupled with Smart-seq2 for precise spatial transcriptomic profiling. *Nat. Commun.* 7:12139. 10.1038/ncomms12139 PMC4941116 27387371

[B40] Nolbrant S. Heuer A. Parmar M. Kirkeby A. (2017). Generation of high-purity human ventral midbrain dopaminergic progenitors for in vitro maturation and intracerebral transplantation. *Nat. Protoc.* 12 1962–1979. 10.1038/nprot.2017.078 28858290

[B41] Panman L. Papathanou M. Laguna A. Oosterveen T. Volakakis N. Acampora D. (2014). Sox6 and Otx2 control the specification of substantia nigra and ventral tegmental area dopamine neurons. *Cell Rep.* 8 1018–1025. 10.1016/j.celrep.2014.07.016 25127144

[B42] Paracchini L. Beltrame L. Boeri L. Fusco F. Caffarra P. Marchini S. (2018). Exome sequencing in an Italian family with Alzheimer’s disease points to a role for seizure-related gene 6 (SEZ6) rare variant R615H. *Alzheimers Res. Ther.* 10:106. 10.1186/s13195-018-0435-2 PMC6182820 30309378

[B43] Parent A. Fortin M. Côtè P. Y. Cicchetti F. (1996). Calcium-binding proteins in primate basal ganglia. *Neurosci. Res.* 25 309–334. 10.1016/0168-0102(96)01065-6 8866512

[B44] Picelli S. Björklund A̧. K. Faridani O. R. Sagasser S. Winberg G. Sandberg R. (2013). Smart-seq2 for sensitive full-length transcriptome profiling in single cells. *Nat. Methods* 10 1096–1098. 10.1038/nmeth.2639 24056875

[B45] Pollen A. A. Nowakowski T. J. Shuga J. Wang X. Leyrat A. A. Lui J. H. (2014). Low-coverage single-cell mRNA sequencing reveals cellular heterogeneity and activated signaling pathways in developing cerebral cortex. *Nat. Biotechnol.* 32 1053–1058. 10.1038/nbt.2967 25086649 PMC4191988

[B46] Poulin J. F. Zou J. Drouin-Ouellet J. Kim K. Y. Cicchetti F. Awatramani R. B. (2014). Defining midbrain dopaminergic neuron diversity by single-cell gene expression profiling. *Cell Rep.* 9 930–943. 10.1016/j.celrep.2014.10.008 25437550 PMC4251558

[B47] Redies C. Hertel N. Hubner C. A. (2012). Cadherins and neuropsychiatric disorders. *Brain Res.* 1470 130–144. 10.1016/j.brainres.2012.06.020 22765916

[B48] Rekaik H. Blaudin de The F.-X. Fuchs J. Massiani-Beaudoin O. Prochiantz A. Joshi R. L. (2015). Engrailed homeoprotein protects mesencephalic dopaminergic neurons from oxidative stress. *Cell Rep.* 13 242–250. 10.1016/j.celrep.2015.08.076 26411690 PMC5066840

[B49] Reyes S. Fu Y. Double K. Thompson L. Kirik D. Paxinos G. (2012). GIRK2 expression in dopamine neurons of the substantia nigra and ventral tegmental area. *J. Comp. Neurol.* 520 2591–2607. 10.1002/cne.23051 22252428

[B50] Schultzberg M. Dunnett S. B. Bjorklund A. Stenevi U. Hokfelt T. Dockray G. J. (1984). Dopamine and cholecystokinin immunoreactive neurons in mesencephalic grafts reinnervating the neostriatum: evidence for selective growth regulation. *Neuroscience* 12 17–32. 10.1016/0306-4522(84)90134-9 6146944

[B51] Simunovic F. Yi M. Wang Y. Macey L. Brown L. T. Krichevsky A. M. (2009). Gene expression profiling of substantia nigra dopamine neurons: further insights into Parkinson’s disease pathology. *Brain* 132 1795–1809. 10.1093/brain/awn323 19052140 PMC2724914

[B52] Sonntag K. C. Song B. Lee N. Jung J. H. Cha Y. Leblanc P. (2018). Pluripotent stem cell-based therapy for Parkinson’s disease: current status and future prospects. *Prog. Neurobiol.* 168 1–20. 10.1016/j.pneurobio.2018.04.005 29653250 PMC6077089

[B53] Studer L. (2017). Strategies for bringing stem cell-derived dopamine neurons to the clinic-The NYSTEM trial. *Prog. Brain Res.* 230 191–212. 10.1016/bs.pbr.2017.02.008 28552229

[B54] Taylor J. M. Macklem M. M. Parsons J. T. (1999). Cytoskeletal changes induced by GRAF, the GTPase regulator associated with focal adhesion kinase, are mediated by Rho. *J. Cell Sci.* 112(Pt 2) 231–242. 10.1242/jcs.112.2.231 9858476

[B55] Thompson L. Barraud P. Andersson E. Kirik D. Bjorklund A. (2005). Identification of dopaminergic neurons of nigral and ventral tegmental area subtypes in grafts of fetal ventral mesencephalon based on cell morphology, protein expression, and efferent projections. *J. Neurosci.* 25 6467–6477. 10.1523/jneurosci.1676-05.2005 16000637 PMC6725273

[B56] Van Hoecke A. Schoonaert L. Lemmens R. Timmers M. Staats K. A. Laird A. S. (2012). EPHA4 is a disease modifier in amyotrophic lateral sclerosis in animal models and in humans. *Nat. Med.* 18 1418–1422. 22922411 10.1038/nm.2901

[B57] Vera E. Bosco N. Studer L. (2016). Generating late-onset human iPSC-based disease models by inducing neuronal age-related phenotypes through telomerase manipulation. *Cell Rep.* 17 1184–1192. 10.1016/j.celrep.2016.09.062 27760320 PMC5089807

[B58] Wang F. Flanagan J. Su N. Wang L. C. Bui S. Nielson A. (2012). RNAscope: a novel in situ RNA analysis platform for formalin-fixed, paraffin-embedded tissues. *J. Mol. Diagn.* 14 22–29. 22166544 10.1016/j.jmoldx.2011.08.002 PMC3338343

[B59] Yamada T. McGeer P. L. Baimbridge K. G. McGeer E. G. (1990). Relative sparing in Parkinson’s disease of substantia nigra dopamine neurons containing calbindin-D28K. *Brain Res.* 526 303–307. 10.1016/0006-8993(90)91236-a 2257487

[B60] Yu Z. L. Jiang J. M. Wu D. H. Xie H. J. Jiang J. J. Zhou L. (2007). Febrile seizures are associated with mutation of seizure-related (SEZ) 6, a brain-specific gene. *J. Neurosci. Res.* 85 166–172. 10.1002/jnr.21103 17086543

[B61] Zhang Y. Chen K. Sloan S. A. Bennett M. L. Scholze A. R. O’Keeffe S. (2014). An RNA-sequencing transcriptome and splicing database of glia, neurons, and vascular cells of the cerebral cortex. *J. Neurosci.* 34 11929–11947. 10.1523/jneurosci.1860-14.2014 25186741 PMC4152602

