# Supplementary material for: Spatial RNA Sequencing Identifies Robust Markers of Vulnerable and Resistant Human Midbrain Dopamine Neurons and Their Expression in Parkinson’s Disease
Source: Front Mol Neurosci. 2021 Jul 8;14:699562. doi: 10.3389/fnmol.2021.699562 (PMC8297217; doi:10.3389/fnmol.2021.699562)
Supplement: Supplementary file 1 [file Data_Sheet_1.pdf]

Spatial RNA sequencing identifies robust markers of vulnerable and resistant human midbrain dopamine neurons and their expression in Parkinson's Disease

Julio Aguila[1,#], Shangli Cheng[3,4#], Nigel Kee[1,2], Ming Cao[1], Menghan Wang[3,4], Qiaolin Deng[3,4, #,*] and Eva Hedlund[1,2,5, #,*]

[1] Department of Neuroscience, Karolinska Institutet, 171 77 Stockholm, Sweden

[2] Department of Cell and Molecular Biology, Karolinska Institutet, 171 77 Stockholm, Sweden

[3] Department of Physiology and Pharmacology, Karolinska Institutet, 171 77 Stockholm, Sweden

[4] Center for Molecular Medicine, Karolinska University Hospital, 171 77 Stockholm, Sweden

[5] Department of Biochemistry and Biophysics, Stockholm University, 106 91 Stockholm, Sweden

[#]Equal contribution

[*]Correspondence: eva.hedlund@dbb.su.se (E.H.) or qiaolin.deng@ki.se (Q.D.)

Table of contents

Supplementary Figure 1

Supplementary Figure 2

Supplementary Figure 3

Supplementary Figure 4

Supplementary Figure 5

Supplementary Figure 6

Supplementary Table 1

Supplementary Table 2

Supplementary Table 3

Supplementary Tables 4 to 9. List of DEGs, Sequencing Stats and Stable Genes (Separate Excel Tables)

Supplementary Table 4. List of differentially expressed genes from previous transcriptomic studies. Related to Supplementary Figure 1.

Supplementary Table 5. List of experimental variables and sequencing stats for the 43 samples associated with the current dataset (18 male subjects). Related to Figures 1, 2 and Supplementary Figure 2.

Supplementary Table 6. List of differentially expressed genes from the current dataset (18 male subjects, 74 DEGs). Related to Figures 1 and Supplementary Figure 2.

Supplementary Table 7. List of differentially expressed genes from the Nichterwitz's dataset [39] re-analyzed here (3 female subjects, 100 DEGs). Related to Figure 1 and Supplementary Figure 2.

Supplementary Table 8. Full list of human stable genes/DEGs ranked by frequency identified with the bootstrapping approach coupled with DESeq2. See also METHODS section. Related to Figures 1, 2 and Supplementary Figure 3.

Supplementary Table 9. Mouse DEGs identified in the La Manno et al., 2016 dataset using the bootstrapping approach coupled with DESeq2. Related to Supplementary Figure 4.

Supplementary Figure 1

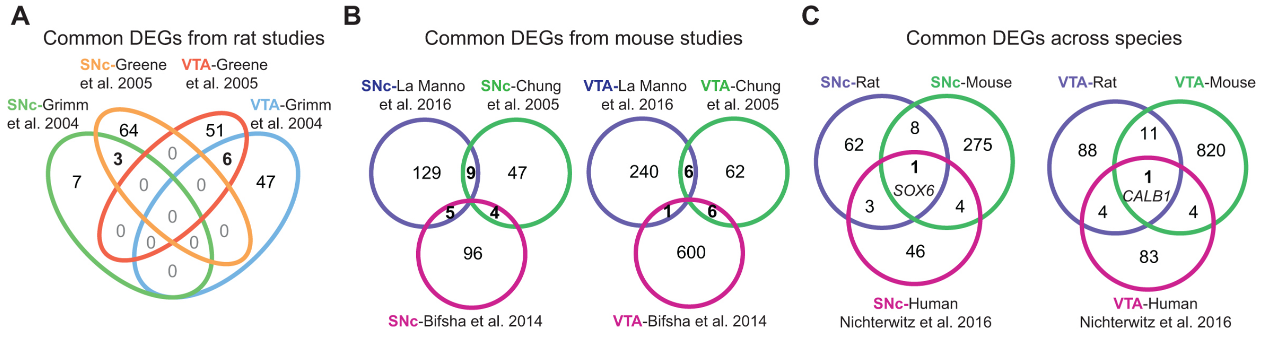

Supplementary Figure 1. Common differentially expressed genes across species. Related to Supplemental Tables S1 and S4. **(A)** Venn diagram showing overlapping DEGs for SNc and VTA identified in previous rat studies (Greene et al., 2005; Grimm et al., 2004). **(B)** Common mouse DEGs are highlighted for SNc and VTA from three different reports (Chung et al., 2005; Bifsha et al., 2014; La Manno et al., 2016). Samples from La Manno et al., 2016 were here analyzed with DESeq2, identifying 390 DEGs. **(C)** Overlapping DEGs between rat, mouse and human (Nichterwitz et al., 2016). For rat and mouse, all previously reported DEGs were considered despite the variability within studies as shown in **(A)** and **(B)**.

Supplementary Figure 2

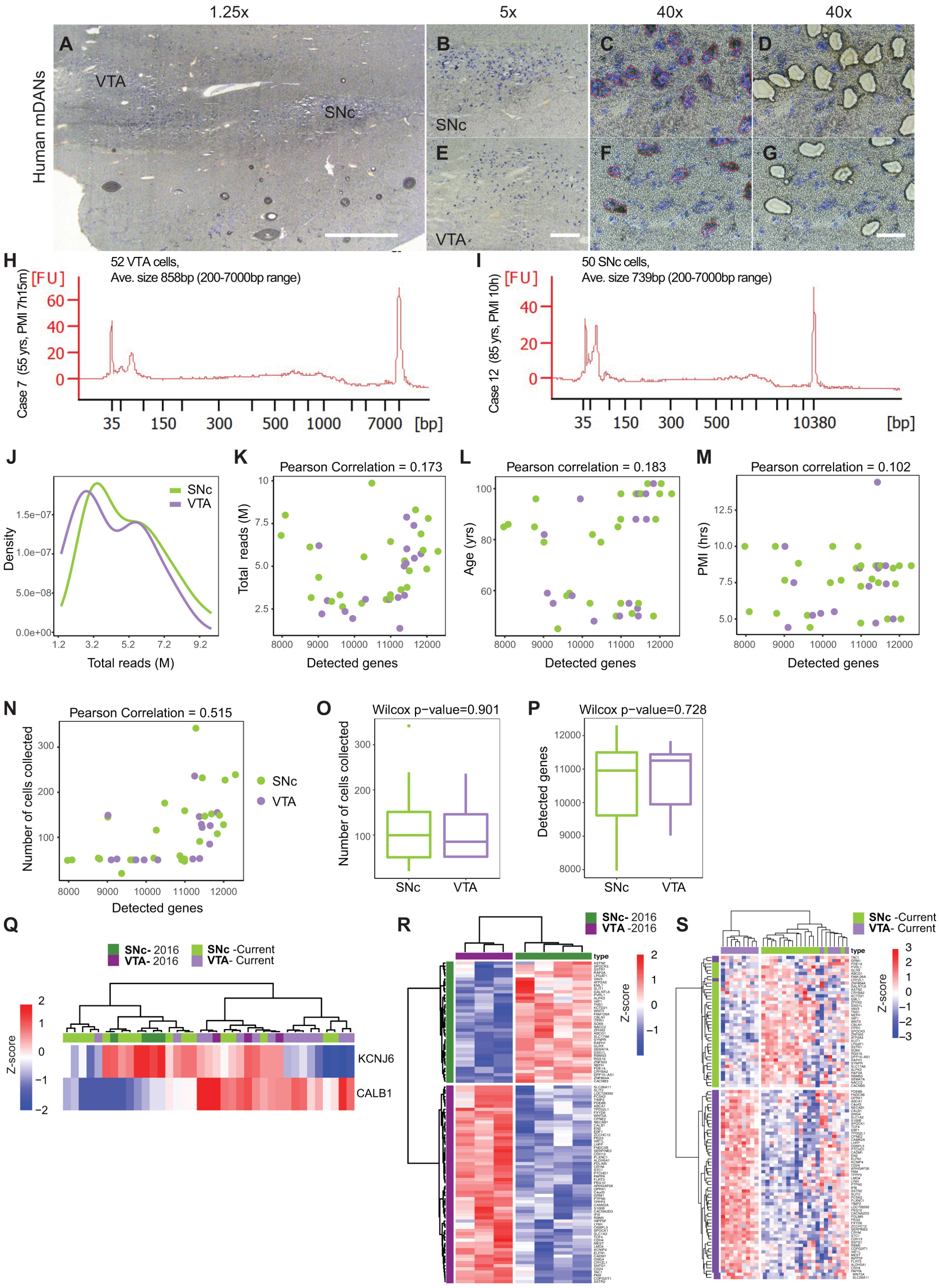

Supplementary Figure 2. Quality control of human adult midbrain dopamine neuron samples profiled using LCM-seq. Related to Figure 1 and Supplemental Tables S2, S5, S6 and S7. **(A** to **G)** Nissl staining of a representative midbrain section for the isolation of SNc **(B,C,D)** or VTA **(E,F,G)** neurons before and after laser capture. **(H, I)**. Representative bioanalyzer profiles of cDNA libraries from VTA **(H)** and SNc **(I)** samples. **(J-P)** The average length size for the cDNA, 700-900 base pairs, was comparable across VTA and SNc. The quality and similarity of samples from both populations was also interrogated based on sequencing stats, including total reads **(J)** and detected genes **(K)**. This later outcome was also correlated to biological and experimental variables such as the "Age" **(L)**, "Post-mortem interval" **(M)** and "Cells" collected during the laser capture sessions **(N)**. **(O, P)** While there was a modest correlation between the number of cells collected and the number of detected genes **(N**, P=0.515**)**, these variables were not significantly different between SNc and VTA groups. See also Supplemental Table S5. **(Q)** Hierarchical clustering of VTA and SNc samples using only *KCNJ6* (*GIRK2*) and *CALB1*. **(R, S)** Samples from Nichterwitz et al., 2016 were re-analyzed with DESeq2 (version 1.16.1) which identified 100-DEGs used to represent those samples **(P,** N=3 subjects**)** or our current dataset **(Q**, N=18 subjects**)**. Scale bars: 500 μm in (A), 200 μm in (B, E), 50 μm in (C,D,F,G).

Supplementary Figure 3

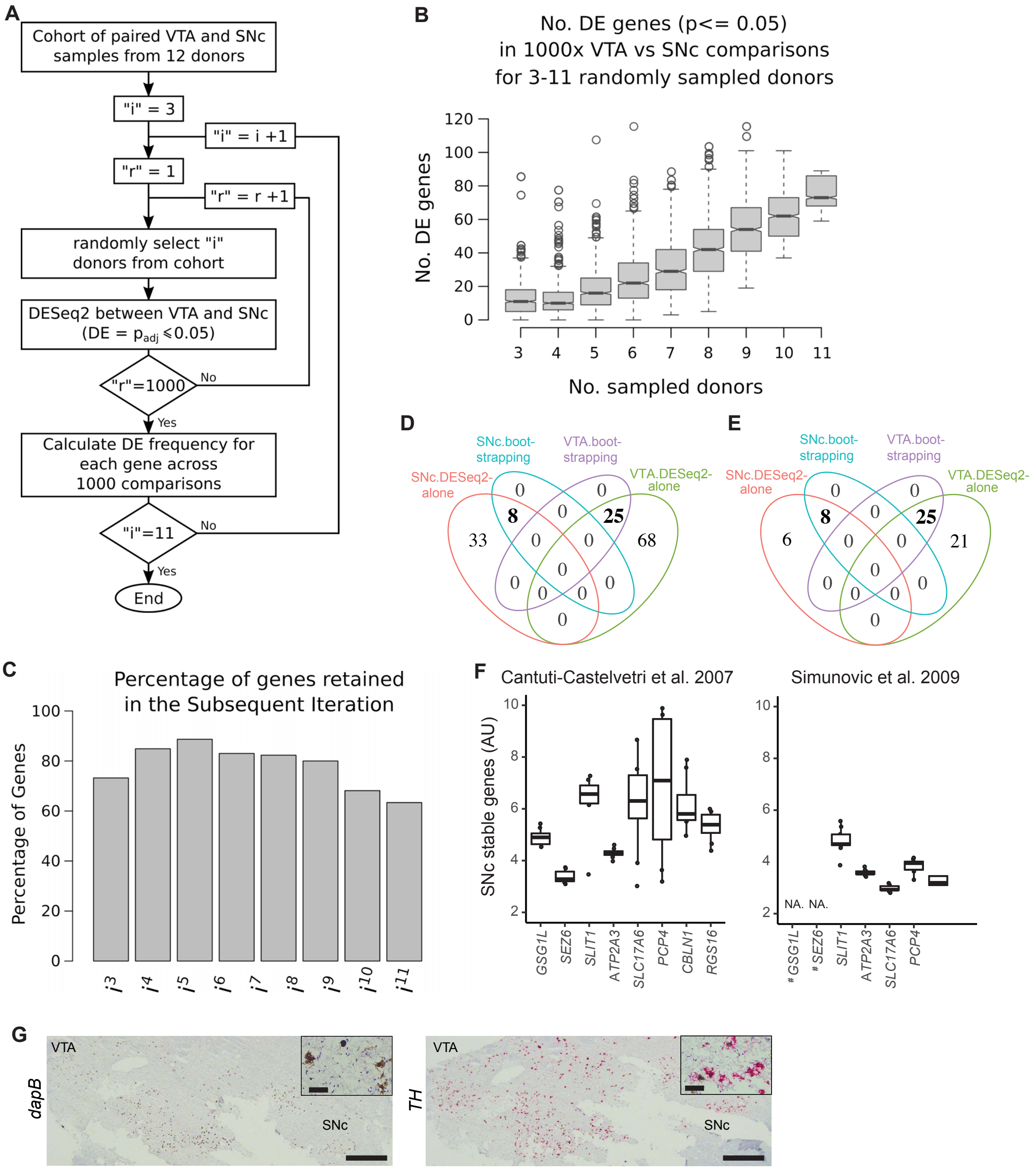

Supplementary Figure 3. Outcome of the bootstrapping approach coupled with DESeq2 applied to human samples and validation of SNc stables genes in the context of previous studies. Related to Figures 1, 2 and Supplemental Tables S2 and S8. **(A)** Flowchart illustrating the computational pipeline for iterative bootstrapping approach, i refers to the sample size of each bootstrapping iteration out of total 12 samples

(3<=i<=11); r refers to iterative times of bootstrapping round in the iteration (r maximum = 1000). **(B)** Boxplot displaying the total number of unique DEGs between VTA and SNc detected in individual iterations. e.g. over 1000x comparisons in i donors. **(C)** Percentage of DEGs in a given iteration, that were retained in the subsequent iterations (3-11 subjects). **(D, E)** To confirm the authenticity of the genes considered stable (33 markers identified through bootstrapping coupled with DESeq2), Venn diagrams were used to assess the overlap with DEGs identified by DESeq2 alone applied to the same 12 subjects (**D**, adj. *P*-value <0.05; E, adj. *P*-value <0.01**)**. **(F)** SNc stable genes are expressed in control human SNc dopamine neurons isolated by LCM from previous microarray studies. [#]In the study reported by Simunovic et al., 2009, the genes *GSG1L* and *SEZ6* were not included in the array. **(G)** RNAscope staining of human melanized midbrain sections using a negative control probe against a bacterial gene (*dapB*) and a positive control probe against tyrosine hydroxylase (*TH*). Scale bars: 1mm in (G) with 50 μm in insets.

Supplementary Figure 4

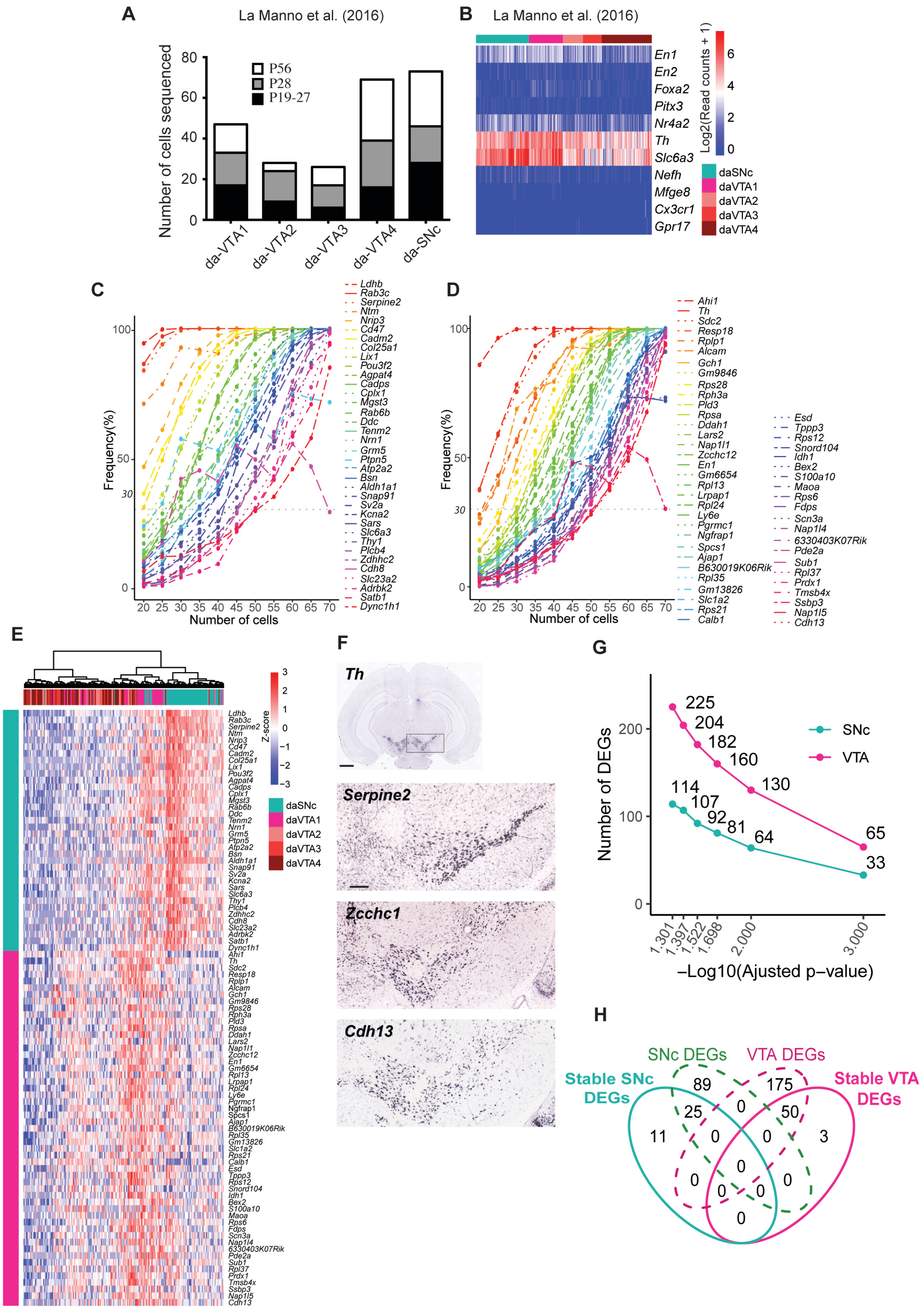

Supplementary Figure 4. Validation and outcome of the bootstrapping approach coupled with DESeq2 applied to a published single cell mouse data set. **(A)** Mouse dataset from La Manno et al., 2016, used to confirm the bootstrapping approach and to identify stable genes in juvenile/adult (postnatal day 19 to 56) midbrain dopamine neurons. The graph shows the number of single cells sequenced for each dopaminergic population. **(B)** Heat map to confirm the expression of known midbrain dopamine neuron markers and the absence of markers for other neural cells as astrocytes, microglia or oligodendrocytes. **(C, D)** Identification of SNc **(A)** and VTA **(B)** stable genes in the mouse dataset from La Manno et al., 2016, using the bootstrapping approach coupled with DESeq2 applied to 146 single cells (73 available SNc cells and 73 randomly selected VTA cells). **(E)** Identified mouse stable genes separate SNc and VTA samples as visualized in the unsupervised clustering. **(F)** *In situ* hybridization images of coronal midbrain sections of the adult mouse (P56, Allen Brain Atlas) showing RNA expression pattern for *Th*, *Serpine2*, *Zcch12* and *Cdh13*. **(G)** The number of DEGs in SNc and VTA from La Manno et al., 2016, identified by DESeq2 as a function of different adjusted P-value cutoffs. **(H)** Venn diagram showing overlaps among stable SNc DEGs, stable VTA DEGs and DEGs in **(G)** with adjust an p-value=0.05.

Supplementary Figure 5

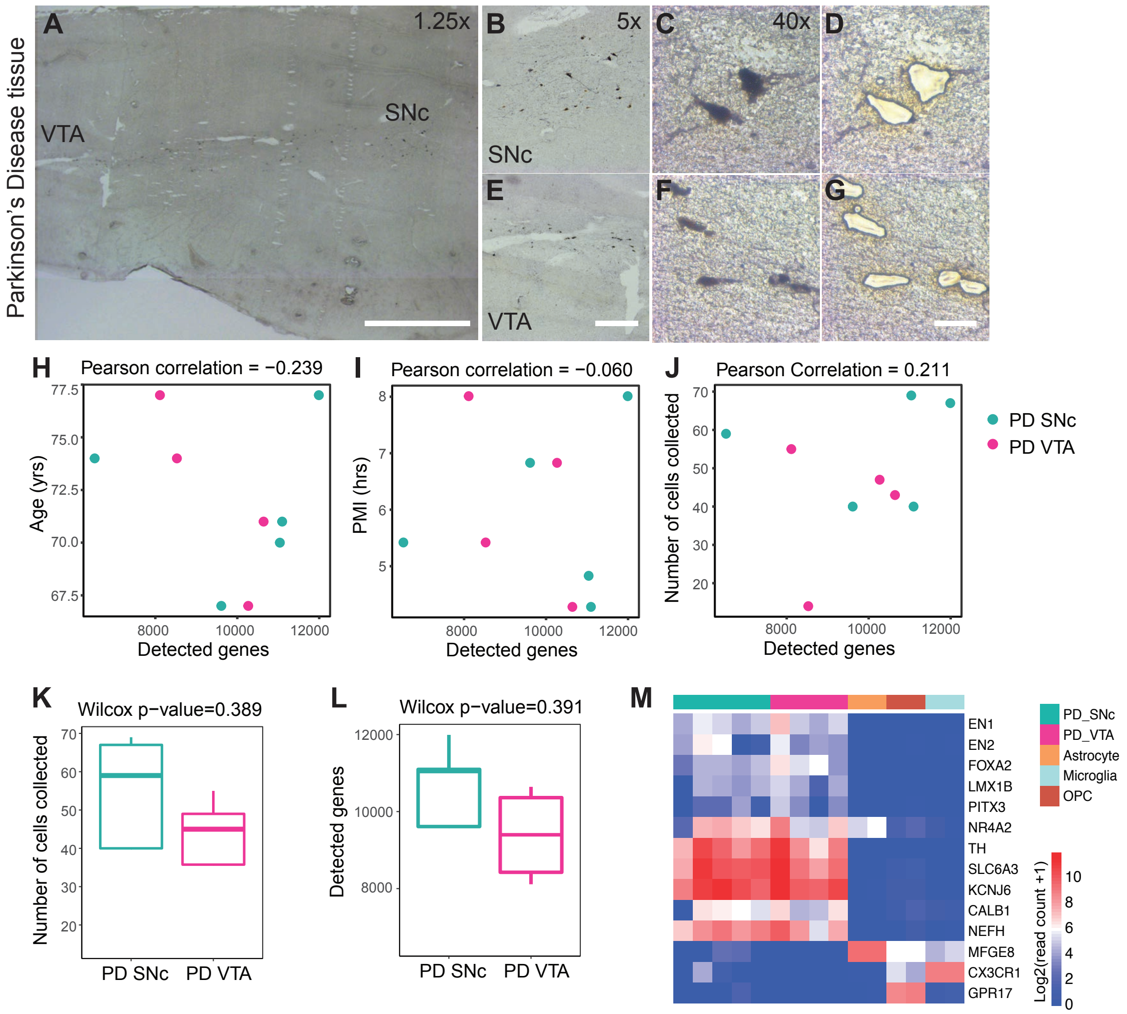

Supplementary Figure 5. Quality control of Parkinson's Disease (PD) patient midbrain dopamine neuron samples profiled using LCM-seq. Related to Figure 5 and Supplementary Table 2. **(A to G)** Nissl staining of a representative midbrain section for the isolation of SNc **(B-D)** or VTA **(E-G)** dopamine neurons from PD patient tissues before and after laser capture. The quality and similarity of samples from SNc and VTA populations were interrogated based on sequencing data and the number of detected genes as a function of "Age" (yrs) of the patients **(H)**, "Post-mortem interval, PMI" (hrs) **(I)** and "The number of cells collected" **(J)** during the laser capture sessions. None of these variables were significantly different between the SNc and VTA groups. Furthermore, the was no significant difference in the number of cells collected for SNc versus VTA **(K)** or the number of detected genes for SNc versus VTA PD samples **(L)**. The high sample quality of the PD samples was confirmed by strong expression of the midbrain dopamine neuron markers *EN1/2*, *FOXA2*, *LMX1B*, *PITX3*, *NR4A2*, *TH* and *SLC6A3* (DAT), the pan-neuronal marker neurofilament (*NEFH*), and the lack of astrocyte, microglia or oligodendrocyte precursor marker contamination, as compared to a published study, Zhang et al., 2014.

Supplementary Figure 6

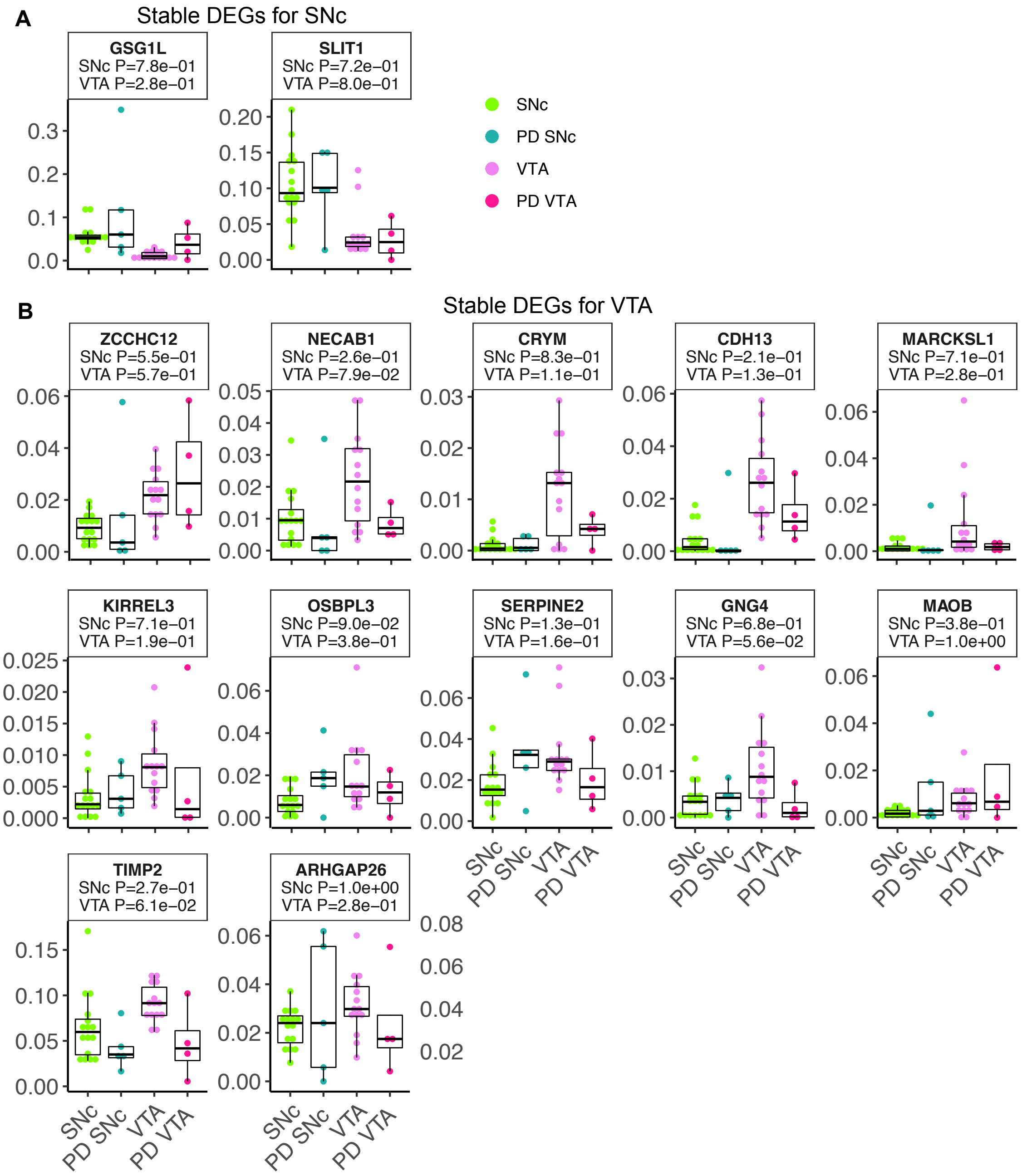

Supplementary Figure 6. Boxplots displaying the expression level of the stable DEGs defining SNc and VTA dopamine neurons from both healthy and PD tissues. **(A)** Two stable DEGs for SNc. **(B)** Twelve stable DEGs for VTA.

Supplementary Table 1. Summary of studies addressing the identity of midbrain dopamine neurons.

Pubmed ID	Sample origin	Sample size	Method	No of DEGs
15353588	Sprague–Dawley rats, adult, female,	>4	qTH stain, LCM bulk, microarray	66
15649693	Lewis rats, adult male	n=8	qTH stain, LCM bulk, microarray	124
15888489	C57/Bl6 mice, adult	n=5-6	qTH stain, LCM bulk, microarray	134
25437550	C57/Bl6 mice, P4P4	mix of three animals	Slc6a3::Cre, FACS-qPCR, single cell array	NA.
25501001	C57/Bl6 mice, P1-P4	duplicates (>2)	TH-EGFP, FACS bulk, microarray	712
27716510	CD-1 mice, juvenile – adult	1-3 brains/time point	FACS and C1 chip, Fluidigm, single cell, RNAseq<br>CD-1, Dat1-Cre/tdTomato)	390 (calculated here)
17412603	Human, adult (male and female)	8 controls	Methylene blue/melanized SNc, LCM bulk, microarray	NA.
19052140	Human, adult, male and female	9 controls	melanized SNc, LCM bulk, microarray	NA.
27387371	Human, adult, female	3 controls	Histogen, LCM-seq bulk, RNAseq	111
Current study	Human, adult (male and female)	18 controls<br>5 PD patients	Histogen, LCM-seq bulk, RNAseq	74

NA. Not applicable.
qTH. Quick tyrosine hydroxylase antibody staining.
FACS. Fluorescence-activated cell sorting
qPCR. Quantitative polymerase chain reaction
LCM. Laser capture microdissection.
RNAseq. RNA sequencing

Supplementary Table 2. Clinical information of subjects used for LCM-seq.

	Gender	Age	Post mortem time	Cause of death	Brain Bank	Targeted cells
1	M	45	8h 50m	Intestinal metastasis	NBB	SNc
2	M	48	5h 30m	Euthanasia, diabetes	NBB	VTA
3	M	50	8h 30m	Cardiac arrest	NBB	SNc and VTA
4	M	51	7h 45m	Suicide, self-withering	NBB	SNc and VTA
5	M	53	14h 25m	Heart failure	NBB	VTA
6	M	55	7h 30m	Euthanasia, cancer	NBB	SNc and VTA
7	M	55	7h 15m	Thrombosis	NBB	SNc and VTA
8	M	58	5h 15m	Thrombosis	NBB	SNc and VTA
9	M	59	4h 25m	Myocardial infarction	NBB	SNc and VTA
10	M	79	7h 40m	Pneumonia and sepsis	NBB	SNc
11	M	82	10h	Pleuritis, cachexia	NBB	SNc and VTA
12	M	85	10h	Prostate cancer, asthma	NBB	SNc
13	M	86	5h 30m	Cancer, cachexia	NBB	SNc
14	M	88	4h 43m	Intestinal bleeding	NBB	SNc and VTA
15	M	88	7h 25m	Euthanasia, cancer	NBB	SNc and VTA
16	M	96	5h 23m	Pneumonia	NBB	SNc and VTA
17	M	98	8h 40m	Cardiac tamponade	NBB	SNc and VTA
18	M	102	5h	Ileus	NBB	SNc and VTA
19	M	67	6h50m	PD, Pneumonia and dehydration	NBB	SNc and VTA
20	M	70	4h50m	PD, Cachexia and dehydration	NBB	SNc
21	M	71	4h17m	PD, Urinary tract infection	NBB	SNc and VTA
22	M	74	5h25m	PD, Euthanasia	NBB	SNc and VTA
23	F	77	8h07m	PD, Pneumonia	NBB	SNc and VTA

M, male
F, female
SNc, substantia nigra compacta
VTA, ventral tegmental area
PD, Parkinson disease (All cases were PD with dementia).
NBB, Netherlands Brain Bank, www.brainbank.nl

SupplementaryTable 3. Clinical information of subjects used for RNAscope.

Case	Gender	Age	Post mortem time	Cause of death	Brain Bank
1	M	48	5h 30m	Euthanasia	NBB
2	M	50	8h 30m	Cardiac arrest	NBB
3	M	55	7h 15m	Intestinal ischemia	NBB
4	M	82	10h 00m	Pleuritis carcinomatosis, cachexia	NBB
5	M	88	4h 43m	Gastrointestinal bleeding	NBB
6	M	98	8h 40m	Cardiac tamponade, atherosclerotic aorta	NBB

M, male
NBB, Netherlands Brain Bank

Supplementary References (all within the reference list of the article)

Bifsha P, Yang J, Fisher RA, Drouin J. 2014. Rgs6 is required for adult maintenance of dopaminergic neurons in the ventral substantia nigra. *PLoS Genet* **10**: e1004863.

Chung CY, Seo H, Sonntag KC, Brooks A, Lin L, Isacson O. 2005. Cell type-specific gene expression of midbrain dopaminergic neurons reveals molecules involved in their vulnerability and protection. *Hum Mol Genet* **14**: 1709-1725.

Greene JG, Dingledine R, Greenamyre JT. 2005. Gene expression profiling of rat midbrain dopamine neurons: implications for selective vulnerability in parkinsonism. *Neurobiol Dis* **18**: 19-31.

Grimm J, Mueller A, Hefti F, Rosenthal A. 2004. Molecular basis for catecholaminergic neuron diversity. *Proc Natl Acad Sci U S A* **101**: 13891-13896.

La Manno G, Gyllborg D, Codeluppi S, Nishimura K, Salto C, Zeisel A, Borm LE, Stott SRW, Toledo EM, Villaescusa JC et al. 2016. Molecular Diversity of Midbrain Development in Mouse, Human, and Stem Cells. *Cell* **167**: 566-580 e519.

Nichterwitz S, Chen G, Aguila Benitez J, Yilmaz M, Storvall H, Cao M, Sandberg R, Deng Q, Hedlund E. 2016. Laser capture microscopy coupled with Smart-seq2 for precise spatial transcriptomic profiling. *Nat Commun* **7**: 12139.

Simunovic F, Yi M, Wang Y, Macey L, Brown LT, Krichevsky AM, Andersen SL, Stephens RM, Benes FM, Sonntag KC. 2009. Gene expression profiling of substantia nigra dopamine neurons: further insights into Parkinson's disease pathology. *Brain* **132**: 1795-1809.

Zhang Y, Chen K, Sloan SA, Bennett ML, Scholze AR, O'Keeffe S, Phatnani HP, Guarnieri P, Caneda C, Ruderisch N et al. 2014. An RNA-sequencing transcriptome and splicing database of glia, neurons, and vascular cells of the cerebral cortex. *J Neurosci* **34**: 11929-11947.
